# Normalization of puberty and adult height in girls with Turner syndrome: results of the Swedish Growth Hormone trials initiating transition into adulthood

**DOI:** 10.3389/fendo.2023.1197897

**Published:** 2023-07-17

**Authors:** Berit Kriström, Carina Ankarberg-Lindgren, Marie-Louise Barrenäs, Karl Olof Nilsson, Kerstin Albertsson-Wikland

**Affiliations:** ^1^ Department of Clinical Science, Pediatrics, Umeå University, Umeå, Sweden; ^2^ Department of Pediatrics, Institute of Clinical Sciences, Göteborg Pediatric Growth Research Center, Sahlgrenska Academy, University of Gothenburg, Gothenburg, Sweden; ^3^ Department of Physiology/Endocrinology, Institute of Neuroscience and Physiology, Sahlgrenska Academy, University of Gothenburg, Gothenburg, Sweden; ^4^ Department of Clinical Sciences, University Hospital Malmö, Lund University, Malmö, Sweden

**Keywords:** adult height, estrogen, growth hormone, height gain, prepubertal growth, pubertal growth, timing of puberty, Turner syndrome

## Abstract

**Objective:**

To study the impact of GH dose and age at GH start in girls with Turner syndrome (TS), aiming for normal height and age at pubertal onset (PO) and at adult height (AH). However, age at diagnosis will limit treatment possibilities.

**Methods:**

National multicenter investigator-initiated studies (TNR 87-052-01 and TNR 88-072) in girls with TS, age 3–16 years at GH start during year 1987–1998, with AH in 2003–2011. Of the 144 prepubertal girls with TS, 132 girls were followed to AH (intention to treat), while 43 girls reduced dose or stopped treatment prematurely, making n=89 for Per Protocol population. Age at GH start was 3–9 years (young; n=79) or 9–16 years (old; n=53). Treatment given were recombinant human (rh)GH (Genotropin^®^ Kabi Peptide Hormones, Sweden) 33 or 67 µg/kg/day, oral ethinyl-estradiol (2/3) or transdermal 17β-estradiol (1/3), and, after age 11 years, mostly oxandrolone. Gain in height_SDS_, AH_SDS_, and age at PO and at AH were evaluated.

**Results:**

At GH start, height_SDS_ was −2.8 (versus non-TS girls) for all subgroups and mean age for young was 5.7 years and that of old was 11.6 years. There was a clear dose–response in both young and old TS girls; the mean difference was (95%CI) 0.66 (−0.91 to −0.26) and 0.57 (−1.0 to −0.13), respectively. The prepubertal gain_SDS_ (1.3–2.1) was partly lost during puberty (−0.4 to −2.1). Age/height_SDS_ at PO ranged from 13 years/−0.42 for GH_67young_ to 15.2 years/−1.47 for GH_33old_. At AH, GH_67old_ group became tallest (17.2 years; 159.9 cm; −1.27 SDS; total gain_SDS_, 1.55) compared to GH_67young_ group being least delayed (16.1 years; 157.1 cm; −1.73 SDS; total, 1.08). The shortest was the GH_33young_ group (17.3 years; 153.7 cm: −2.28 SDS; total gain_SDS_, 0.53), and the most delayed was the GH_33old_ group, (18.5 years; 156.5 cm; −1.82 SDS; total gain_SDS_, 0.98).

**Conclusion:**

For both young and old TS girls, there was a GH-dose growth response, and for the young, there was less delayed age at PO and at AH. All four groups reached an AH within normal range, despite partly losing the prepubertal gain during puberty. Depending on age at diagnosis, low age at start with higher GH dose resulted in greater prepubertal height gain, permitting estrogen to start earlier at normal age and attaining normal AH at normal age, favoring physiological treatment and possibly also bone health, hearing, uterine growth and fertility, psychosocial wellbeing during adolescence, and the transition to adulthood.

## 1 Introduction

The main characteristics of Turner syndrome (TS), a sex-chromosomal pathology syndrome, are short stature and gonadal failure. The etiology of short stature is multifactorial and may depend partly on haplo-insufficiency of the *SHOX* gene (1, 2). Growth for girls with TS is reduced in all phases of growth, being fetal–infancy, childhood, and puberty, compared to non-TS girls growth models (3, 4) and references (5, 6). As a result, TS is associated with an adult height (AH) approximately 20 cm below that predicted based on mid-parental height (MPH). Research from adult women with TS has shown that having an AH within the normal range and undergoing puberty at a normal time relative to their peers are of great importance for quality of life (QoL) (7, 8). For this reason, growth-promoting and puberty-inducing therapies have been used in girls with TS for many years.

Androgens were used as growth promoter even before recombinant human (rh) growth hormone (GH) was approved in 1986 (9). In Sweden by 1986, girls with TS aged 9 years or older were included in national investigator-initiated multicenter trials of rhGH (33 µg/kg/day) and estrogen replacement therapy (ERT) (10). These studies were in 1987 expanded to include three investigator-initiated trials of rhGH treatment also including girls from 3 years of age (4). From 1991, the rhGH dose was increased in line with the upper dose used in subsequent GH-trials for optimizing pubertal growth in GH deficiency (GHD) and for other possible indications (67 µg/kg/day) (9, 11–13). Such a temporal study design allowed the assessment of dose–response (14). It should also be possible to evaluate both growth response and GH responsiveness during the first year of treatment (15) and with individualized growth prediction models (16, 17) by using the one for TS (18).

What have we learned about growth in girls with TS since the first trials were initiated more than 35 years ago? Growth-promoting treatment with rhGH at different doses and with or without ERT and androgens in girls with TS has shown varying results both in short-term studies (14, 19, 20) and long-term studies to AH (21) undertaken in different countries (10, 22–31) and from worldwide outcome databases as pioneering KIGS (32–36).

Today, there is consensus that, if age at diagnosis permits, puberty should be induced at a “normal age” in girls with TS (37). Due to the known physiological effects of estrogen on most tissues and organ systems, puberty should ideally be induced using a dosage regimen that mimics the increasing serum estradiol levels observed in normal female puberty (38, 39), and girls should subsequently be maintained on a dose that results in serum levels appropriate for young adult women, which is twice that recommended for postmenopausal women (40–42), considering uterine size with fertility aspects (43, 44), future bone (45), and cardiovascular health (46, 47).

The goal for girls with TS in the 1980s, as it is now, was to normalize height during childhood so that puberty could be induced within the normal age range, allowing for normal tempo of the progress of subsequent pubertal growth and maturation and the attainment of a normal AH within the expected normal age range. To achieve this, it is necessary to balance projected height with age at puberty induction, using incremental doses of ERT. This is further complicated by delays in the age at diagnosis of TS, which can limit the time available for growth-promoting treatment. The key question addressed in the present analysis was what difference would there be when starting treatment in the young girl compared with that in an older girl using rhGH, oxandrolone, and estrogen? This is now investigated in the present study, using long-term data from the above-mentioned investigator-initiated multicenter trials conducted in Sweden between 1987 and 2011, which followed 132 girls with TS to AH, partly presented at the fifth TS meeting (14) and at ESPE (48).

## 2 Material and methods

### 2.1 Ethics

The trials (TRN 87-052-01 and TRN 88-072) were approved by the Ethical Committees of Sweden at the university hospitals in Lund (221/87), Göteborg, Linköping, Umeå, Uppsala (all 76-88), and the Karolinska Institute (88–40). National approval for the last part of the study was received from Lund (400/91). Informed consent was obtained from the parents and from the girls if they were old enough to understand.

### 2.2 Study subjects

#### 2.2.1 Inclusion criteria

The study included girls with TS (karyotype from at least 25–30 lymphocytes) aged between 3.0 and 15.9 years and with height standard deviation scores (SDSs) below –1 compared with the reference population of healthy Swedish girls born approximately the same years (6). All Turner karyotypes were accepted, except for those associated with a Y-chromosome cell line. Girls with normalized thyroid function and moderately well-treated epilepsy were accepted.

#### 2.2.2 Exclusion criteria

Girls were excluded if they (1) had severe diseases including coeliac disease; (2) had been previously treated with GH, sex hormones, or corticosteroids; (3) had a Turner karyotype containing a Y-chromosome cell line; or (4) they were unable to attend.

#### 2.2.3 Intention to treat population

**Figure 1:**
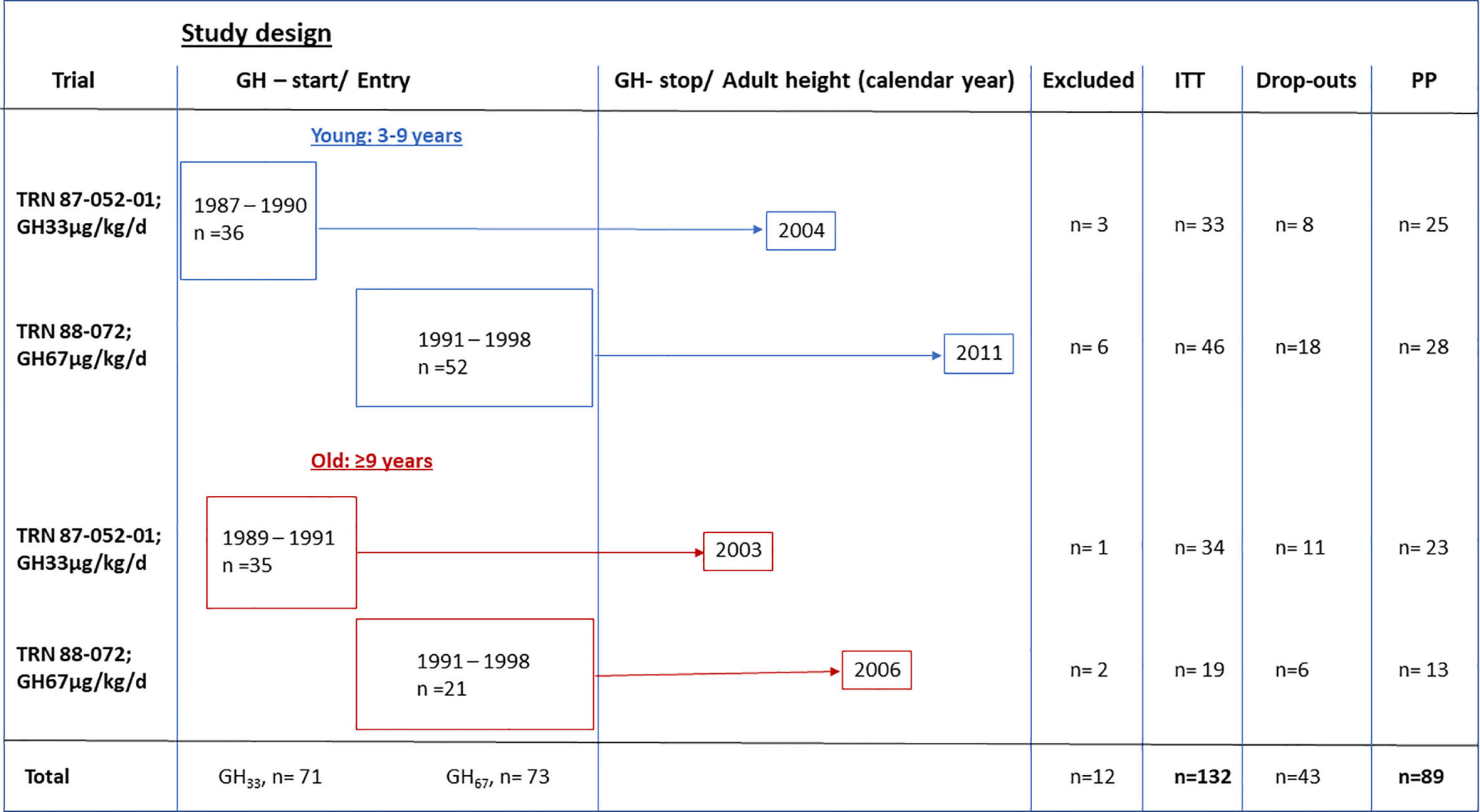
Study design for the GH trials of the temporal designed study. Calendar year at enrollment and GHstart and GHstop and adult height indicated, as excluded from intention to treat (ITT) and dropouts from per protocol (PP) population.

Between 1987 and 1998, 144 girls with TS who were naive to GH treatment were enrolled in Swedish multicenter studies in accordance with the criteria above; they received GH at a dose of 33 or 67 µg/kg/day (see study design), either alone or alongside treatment with oxandrolone and estrogen, depending on their age. Girls starting GH treatment from 1987 to 1991 received the 33 µg/kg/day dose, while girls starting treatment from 1991 onwards received 67 µg/kg/day. At GH treatment start, 88 girls were 3–9 years old, and 56 were over 9 years of age; 67 girls received 33 µg/kg/day, and 65 received 67 µg/kg/day doses. Overall, 132 of the 144 girls were followed to AH and constituted the intention-to-treat (ITT) population (**Figure 1**). Girls were assigned to subgroups based on age and GH dose. After enrollment, there were 12 girls not followed to AH, two were excluded owing to missing data and five owing to the initiation of GnRH-analogue treatment in TS girls with spontaneous puberty. Five developed other diseases after enrollment: two with coeliac, two with epilepsy, and one with high blood sugar, however, not diabetes mellitus.

#### 2.2.4 Per protocol population

The per protocol (PP) population constituted 89 girls (**Figure 1**). Protocol violations occurred in 43 girls; these included significant GH dose reduction or premature GH treatment cessation when the girl was satisfied with her height (n=35 with height velocity more than 2.5 cm/year) or before AH was attained (n=8 with another 2.2–7.9 cm until AH) (**Figure 1**).

#### 2.2.5 Pretreatment characteristics

The pretreatment characteristics of the four ITT study subgroups are shown for the ITT population in **Table 1** for the PP population and in **Supplementary Tables S1** and **S2**. Karyotype 45,X was found in 73 girls, mosaicism in 5 girls, and structural abnormalities in 53 girls.

**Table 1A:** Pre-treatment characteristics for the young groups 3—9 years at GH start in the ITT population versus normal population (6, 49, 50).

Variables	Dose 33 µg (n=33)	Dose 67 µg (n=46)	p-value	Difference between groups Mean (95% CI)	Effect size
**Karyotype 45X**	25 (80.6%)	25 (54.3%)			
**Mosaic**	1 (3.2%)	2 (4.3%)			
**Other**	5 (16.1%)	19 (41.3%)	0.051		
**Karyotype missing**	2	0			
At birth					
**GA** **(weeks)**	38.6 (1.8) 39 (34; 42) n=33	38.5 (2.0) 39 (33; 42) n=46	0.86	0.095 (−0.784; 0.981)	0.049
**Length** **(SDS)**	−2.53 (1.41) −2.68 (−5; 0.9) n=33	−1.86 (1.17) −1.78 (−4.34; 0.23) n=46	0.023	−0.670 (−1.242; −0.098)	0.525
**Weight** (SDS)	−1.61 (1.41) −1.39 (−5.11; 0.37) n=33	−1.12 (1.22) −1.1 (−3.92; 1.7) n=46	0.11	−0.489 (−1.080; 0.104)	0.375
**Mother height** **(SDS)**	−0.23 (0.94) −0.18 (-2.73; 1.3) n=33	0.01 (1.21) -0.10 (-2.09; 2.63) n=46	0.35	−0.232 (−0.734; 0.265)	0.210
**Father height** **(SDS)**	−0.21 (0.90) −0.26 (-1.79; 1.70) n=33	−0.14 (1.10) 0.10 (-3.33; 1.74) n=46	0.77	−0.069 (−0.529; 0.395)	0.067
**MidParental Height** **(SDS)**	−0.27 (0.90) −0.44 (-2.05; 1.86) n=33	−0.08 (1.10) -0.19 (-3.36; 2.07) n=46	0.42	−0.187 (−0.650; 0.276)	0.183
**DiffMPH** **(SDS)**	−2.26 (1.71) −2.17 (−5.79; 1.48) n=33	−1.78 (1.32) −1.83 (−4; 0.93) n=46	0.15	−0.483 (−1.162; 0.191)	0.323
**Pre-treatment height velocity, before GHstart (cm/year)**	5.49 (1.73) 5.4 (3.14; 12.3) n=33	5.54 (1.45) 5.47 (2.51; 9.48) n=45	0.89	−0.053 (−0.774; 0.657)	0.034

For categorical variables n (%) is presented.

For continuous variables, mean (SD)/median (Min; Max)/n= is presented.

For comparison between groups, chi-square exact test was used for non-ordered categorical variables, and the Fisher’s non-parametric permutation test was used for continuous variables. The confidence interval for the mean difference between groups is based on Fisher’s non-parametric permutation test.

Effect size is absolute difference in mean/pooled SD.

GA, gestational age; GH, growth hormone; SDS, standard deviation score; MPH, mid-parental height; DiffMPH, difference in SD score between the height of the girl and the heights of her parents.

#### 2.2.6 Laboratory analyses for diagnostic and safety purposes

*GH–IGF axis*. Before the start of treatment, a 24hGH profile was obtained with 30 min sampling, and serum IGF-1, IGFBP1, and IGFBP3 were determined. IGF-1 and IGFBP3 were determined again after 10, 30, and 40 days, and thereafter at the yearly visit (51). GHBP was also analyzed (52).

*TSH–thyroxine axis*. TRH, TSH, free thyroxin fT4, and fT3 were determined before GH treatment start and every 6 months thereafter: TSH, fT4, and fT3 (53)

*Gonadal axis*. FSH, LH, DHEAS, androstenedione, estradiol, and SHBG were analyzed before GH treatment start and yearly thereafter. LHRH was analyzed yearly from 5 years of age onwards; FSH forms were explored (54).

*Glucose metabolism*. Intravenous glucose tolerance test, b-glucose, HbA1c, and urine test for protein and glucose were conducted at treatment start and yearly thereafter. HbA1c was analyzed every 6 months, and urine was tested for protein and glucose every 3 months.

*Coeliac disease*. Gliadin antibody test was performed (55).

*Blood status*. Hb, LPK, Na, K, urea, and ALP were analyzed every 6 months.

*GH antibody analysis*. GH antibody analysis was performed.

### 2.3 Study design

**Figure 2:**
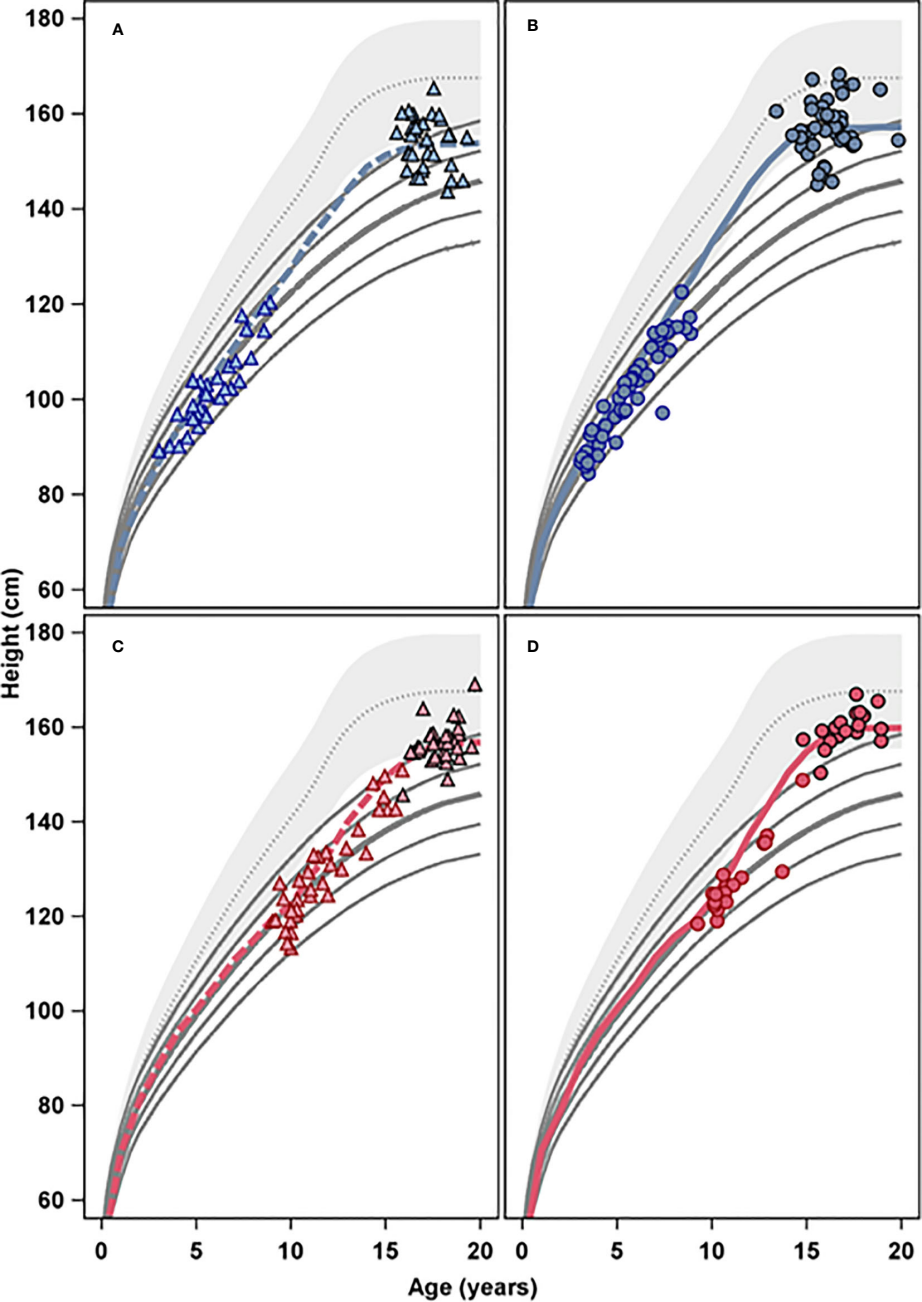
The four panels present height (cm) versus age (years) for the four different age and treatment groups, GH _33 young_ **(A)**, GH _67 young_ **(B)**, GH _33 old_ **(C)**, and GH _67 old_ **(D)**; open symbols represent GH start and filled symbols represents adult height. The colored lines represent mean growth for the four treatment groups, blue for the young groups, and red for the old groups, respectively, in relation to height reference from the healthy girls (gray area) (6) and from girls with Turner Syndrome (solid lines) (5).

A total of 144 girls were included consecutively and were assigned to four subgroups based on age at diagnosis (3–9 vs. >9–15.9 years) and GH dose (33 vs. 67 µg/kg/day) at treatment start. Before any analysis, data from 12 girls were excluded, 5 due to LHRH agonist treatment because of short stature at age of onset of spontaneous puberty. At GH treatment start, the ITT population included 79 girls aged 3–9 years (young group) and 53 girls aged over 9 years (old group). The 67 girls who started GH treatment before mid-1991 received 33 µg/kg/day dose, and the 65 who started treatment after this point received 67 µg/kg/day dose (**Figures 1**, **2**). The 132 girls were assigned to groups as follows:

GH_33young_ (n=33): girls were enrolled 1987–1990 (Turner III) and started treatment with 33 µg/kg/day GH aged 3–9 years; the last girl reached AH in 2004.
GH_33old_ (n=34): girls were enrolled 1989–1991 (Turner IV) and started treatment with 33 µg/kg/day GH aged >9 years; last girl reached AH in 2003.
GH_67young_ (n= 46): girls were enrolled 1991–1998 (Turner V) and started treatment with 67 µg/kg/day GH at 3–9 years; last girl reached AH in 2011.
GH_67old_ (n=19): girls were enrolled 1991–1998 (Turner V) and started treatment with 67 µg/kg/day GH aged >9 years; last girl reached AH in 2006.

### 2.4 Hormonal treatment

#### 2.4.1 GH treatment

RhGH [Genotropin^®^, Kabi Peptide Hormones (Turner III–IV), Kabi Pharmacia Corp. (Turner V), Stockholm, Sweden] 33 or 67 µg/kg was injected deep subcutaneously every evening (56). Girls on the 67 µg/kg/day dose were started on 33 µg/kg/day; the dose was increased stepwise over a period of 1–6 months to avoid water retention and edema. GH dose was adjusted to body weight every 3 months. GH treatment was stopped when growth velocity fell below 2 cm/year or when the girl was satisfied with her height (n=43).

#### 2.4.2 Oxandrolone treatment

Oxandrolone (Anavar^®^, Searle Ltd., Chicago, USA) 0.05 mg/kg/day was allowed according to the protocol from 11 years of age (bone age ≥9 years) if growth velocity and/or height criteria were not satisfied despite good compliance according to the investigator’s clinical judgment. When initiated during childhood, oxandrolone treatment was used until AH in 54% of the young girls, and among the older girls, 94% had started oxandrolone 1–2 years after GH start, i.e., before the start of estrogen replacement therapy (puberty onset).

#### 2.4.3 Estrogen replacement therapy

##### 2.4.3.1 Oral ethinyl-estradiol

During the 1980s and early 1990s, puberty was initiated using oral ethinyl-estradiol (EE2) (Etivex^®^, Leo Pharma Corp., Malmö, Sweden). Age at treatment initiation was at the investigator’s discretion; the intention was to start treatment with a dose of 25 ng/kg/day at an appropriate annual visit when the patient was close to 13 years of age. For the first year, the dose was increased by 25 ng/kg/day every 3 months; thereafter, there was a yearly dose increment of 100 ng/kg/day. The older girls with gonadal failure, group GH_33old_ and GH_67old_, started EE2 at 13–14 years of age with the same starting dose. For girls >14 years, wishing for a more rapid pubertal development, a starting EE2 dose of 100 ng/kg/day was allowed with yearly dose increments of 100 ng/kg/day. Gestagen (Medroxi-progesterone, Gestapuran^®^, Leo Pharma Corp., Malmö, Sweden) was added when EE2 dose reached 300 ng/kg/day.

##### 2.4.3.2 Transdermal 17β-estradiol

In 1997, ERT was changed to transdermal 17β-estradiol for most girls. At that time, starting dose was a 5-µg patch (Estraderm^®^ Serono, Schweiz), and from 2001, this became a 6.25–12.5-µg patch (1/4–1/2 part of the matrix patch Evorel^®^ (=Systen^®^) 25 µg/24 h; Janssen-Cilag Pharmaceutica N.V, Beerse, Belgium), corresponding to 0.13 ± 0.03 µg/kg bodyweight with application of the piece of patch initially only at nighttime (38). Girls were maintained on the initial dose for 9 months, with the aim of inducing breast development. Thereafter, the dose was increased by 6.25 µg every 6 months until what was then considered to be the adult regimen was reached (a continuous 25-µg patch, changed twice/week). Gestagen was added approximately 2 years after the start of ERT.

### 2.5 Monitoring

Height (mean out of three measures using a Harpenden stadiometer), sitting height, and weight were measured at baseline and thereafter every 3 months. AH was considered to have been achieved at the time when growth velocity was below 1 cm/year. Pubertal maturation was assessed according to breast development Tanner stage 1–5.

## 3 Methods

### 3.1 Growth evaluation

#### 3.1.1 Using references of healthy girls

Birth length and weight were converted to SD scores (SDS) relative to a reference population of ~800,000 healthy newborns born in Sweden from 1990 to 1999 (49). All measurements were corrected for gestational age. Height at start of GH treatment and the last recorded height before puberty (prepubertal height) were converted to SDS using the childhood component (3) applied to the Swedish reference population born in 1974 (6), to calculate gain in height_SDS_ during the prepubertal and pubertal period.

The difference from current height_SDS_ to mid-parental height (MPH) SDS is referred to as diff_SDS_. Diff_SDS_ was calculated at different time points including GH treatment start, the start of puberty, and at AH. MPH_SDS_ was calculated as: (father’s height_SDS_ + mother’s height_SDS_)/1.61 (50).

AH was measured in centimeters and converted into SDS with heights transferred to “age 18 years for AH” for the reference population, irrespective of actual age at AH (6).

GH efficacy, i.e. height gain, during the childhood growth period (prepuberty), is given in SDS according to the childhood function from the ICP model (3) applied in the used reference (6). Gain in centimeters from GH start until onset of puberty (last recorded prepubertal height) is not included, as it only reflects time from treatment start. Height gain during puberty is calculated in centimeters and by calculating AH_SDS_ minus height_SDS_ at last prepubertal visit. Total gain in height_SDS_ was calculated using AH_SDS_ minus height_SDS_ at GH start.

Duration of puberty was calculated based on the difference between age at the last prepubertal visit or at the visit when ERT was started and the age when AH was attained.

#### 3.1.2 Using reference for girls with Turner syndrome

All lengths and heights were also converted to SDS relative to a reference for girls with TS obtained from data on spontaneous untreated growth in girls with TS in Sweden, Denmark, and the Netherlands (5), and versus the Childhood component of the Turner ICP-growth model, used here for calculating height gain during puberty (4).

### 3.2 Statistical analyses

Continuous variables were described using the mean, SD, median and range, and categorical variables using n and %.

For comparison between the two groups, Fisher’s exact test was used for dichotomous variables, A chi-squared test was used for non-ordered categorical variables, a Mantel–Haenszel chi-squared test was used for ordered categorical variables, and Fisher’s non-parametric permutation test for comparison of two means was used for continuous variables. The main results from the comparison between two groups regarding dichotomous and continuous variables were presented as mean difference with 95% confidence interval (CI). For continuous variables, effect size between the two groups was also given. Effect size was defined as mean difference/pooled SD.

A forward stepwise linear regression was used to select independent predictors for each outcome variable. Only those predictors with a univariate relationship with p<0.1 to each outcome variable were included as possible predictors. The explained variance (r²) was calculated for each model, together with beta-coefficient with 95% CI for each predictor, i.e., independent variable.

All tests were two-tailed and conducted at the 5% significance level.

## 4 Results

### 4.1 GH-dose dependency of height outcomes

**Figure 3:**
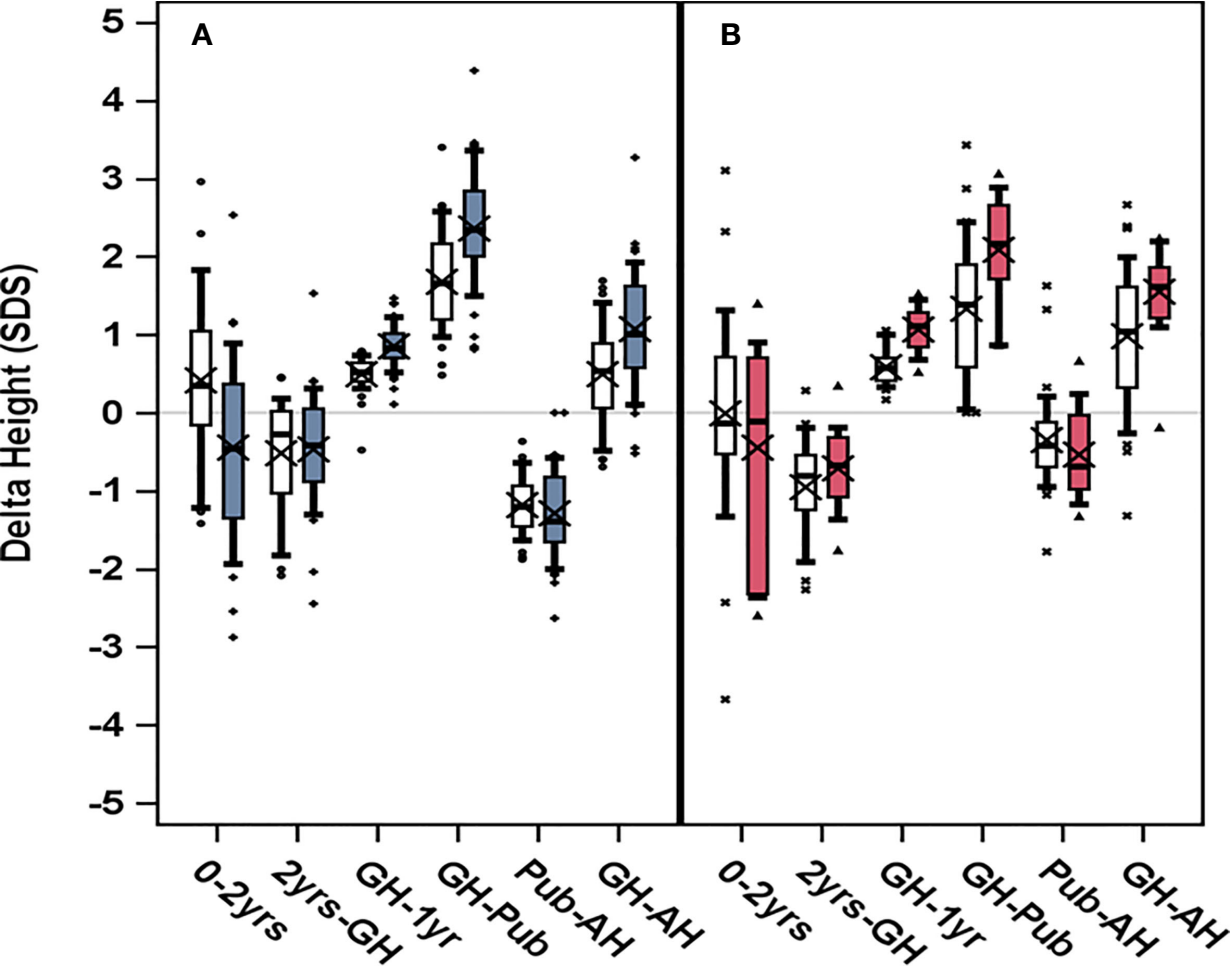
Change in height_SDS_, delta height, trough different growth periods for girls who started GH treatment at **(A)** age 3–9 years to the left, blue, or **(B)** after age 9 years to the right, red. GH dose 33 μg/kg/day are depicted in light color boxes; dark color boxes (blue for young and red for old) represent GH dose 67 μg/kg/day. Boxplots show 5th, 25th, 50th, 75th, and 95th percentiles, and “X” represents the group mean value. Height_SDS_ was calculated in relation to reference from non-TS girls (6), for infancy (0–2 years), for childhood (2 years GH) as pretreatment growth, for prepubertal GHstart to onset of puberty (GH-Pub) as prepubertal gain, for onset of puberty to adult height (Pub-AH) as pubertal gain, and for the total period on GH (GH-AH) as total gain.

**Figure 4:**
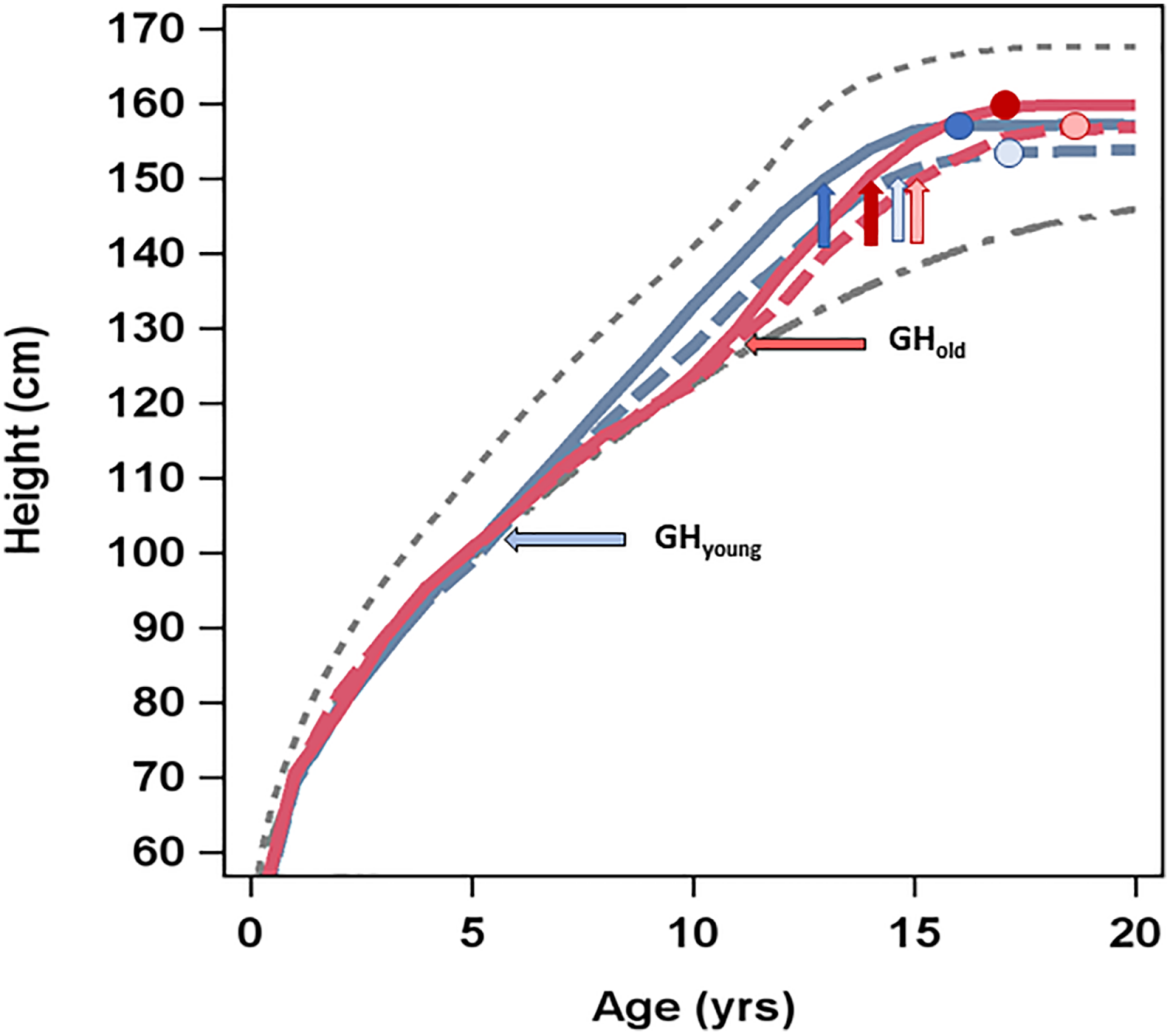
Average height (cm) over time by age (years) at GH start and GH dose. The upper (dotted) line presents average height over time in healthy girls (6), the lower (dashed) line shows the average height over time in untreated girls with Turner syndrome (5), and the four lines in between present the four treatment groups: GH_33young_ (blue dotted line), GH_67young_ (blue solid line), GH_33old_ (red dotted line), and GH_67old_ (red solid line). Horizontal arrows depict age (years) at GH start for the different groups. Vertical arrows depict age at puberty onset, and circles show height and age at attained adult height.

**Figure 5:**
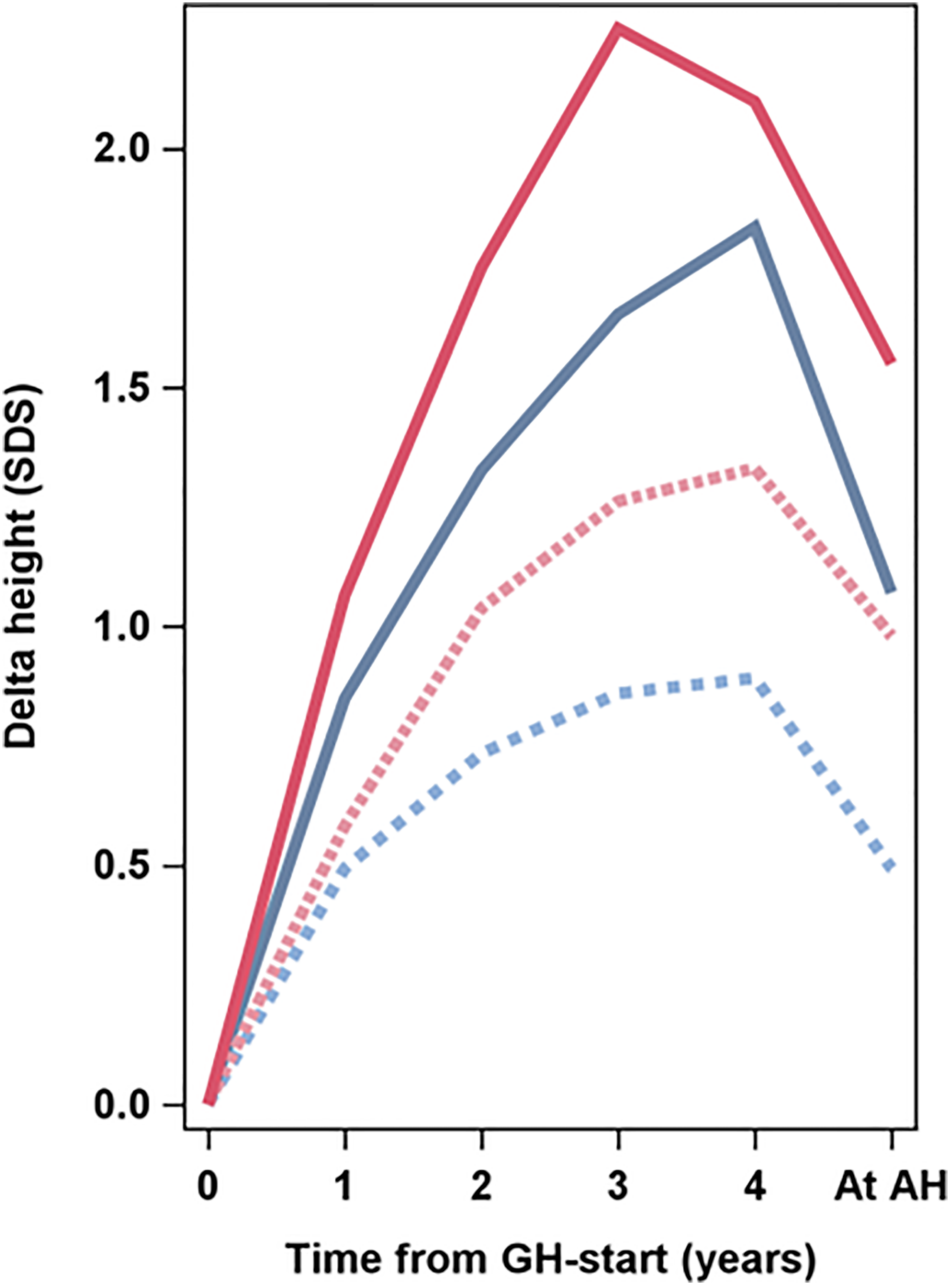
Average delta height_SDS_ in relation to Swedish reference for healthy girls (6) versus time from start of GH treatment to adult height (AH) (years). The four lines present the four groups: GH_33young_ (blue dotted line), GH_67young_ (blue solid line), GH_33old_ (red dotted line), GH_67old_ (red solid line). The Swedish reference used for SDS (6).

A clear GH-dose response effect was found in girls classified as young or as old at time of GH start, both in the ITT and the PP population (**Figures 3**, **4**, **5**). For the ITT population, the progress in height_SDS_ from birth to AH, for three comparisons, is presented: for pre-treatment characteristics, young in **Table 1A** and old in **Table 1B**,

**Table 1B:** Pre-treatment characteristics for the old groups ≥9 years at GH start in the ITT population, SDS versus Swedish references (6, 49, 50).

Variables	Dose 33 µg (n=34)	Dose 67 µg (n=19)	p-value	Difference between groups Mean (95% CI)	Effect Size
**Karyotype 45X**	13 (38.2%)	10 (52.6%)			
**Mosaic**	2 (5.9%)	0 (0.0%)			
**Other**	19 (55.9%)	9 (47.4%)	0.42		
**Missing**	0	0			
At birth					
**GA** **(weeks)**	39.3 (1.4) 39 (36; 42) n=33	38.2 (1.8) 38 (34; 41) n=17	0.033	1.10 (0.11; 2.00)	0.699
**Length** **(SDS)**	−1.98 (1.40) −1.81 (−5.49; 0.97) n=34	−1.81 (1.24) −1.67 (−4.48; 0.31) n=18	0.68	−0.167 (−0.953; 0.606)	0.124
**Weight** **(SDS)**	−1.49 (1.08) −1.24 (−4.73; 0.42) n=34	−1.13 (1.08) −0.98 (−3.07; 1.41) n=19	0.25	−0.359 (−0.983; 0.252)	0.332
**Mother height** **(SDS)**	−0.27 (0.70) −0.18 (−1.46; 1.09) n=33	−0.04 (0.88) −0.18 (−1.78; 1.73) n=19	0.31	−0.227 (−0.672; 0.223)	0.295
**Father height** **(SDS)**	0.03 (0.83) 0.05 (−1.71; 1.82) n=31	0.17 (0.87) 0.14 (−1.79; 1.59) n=19	0.60	−0.135 (−0.624; 0.362)	0.160
**MPH** **(SDS)**	−0.16 (0.73) −0.09 (−1.50; 1.23) n=31	0.08 (0.82) 0.00 (−1.55; 1.78) n=19	0.29	−0.241 (−0.691; 0.210)	0.315
**DiffMPH** **(SDS)**	−1.89 (1.43) −1.71 (−5.89; 0.96) n=31	−1.98 (1.37) −1.87 (−4.4; 0.69) n=18	0.83	0.091 (−0.750; 0.921)	0.064
**Pretreatment height velocity, before GHstart (cm/year)**	3.51 (1.05) 3.54 (1.14; 5.32) n=34	3.79 (0.63) 3.9 (2.82; 4.75) n=16	0.33	−0.278 (−0.846; 0.277)	0.297

For categorical variables, n (%) is presented.

For continuous variables, mean (SD)/median (Min; Max)/n= is presented.

For comparison between groups chi-square exact test was used for non-ordered categorical variables, and the Fisher’s non-parametric permutation test was used for continuous variables.

The confidence interval for the mean difference between groups is based on Fisher’s non-parametric permutation test.

Effect size is absolute difference in mean/pooled SD.

GA, gestational age; GH, growth hormone; SDS, standard deviation score; MPH, mid-parental height; DiffMPH, difference in SD score between the height of the girl and the heights of her parents.

A: for comparisons between GH dose within each age group, young, in **Tables 2A**, **3A** and old in **Tables 2B**, **3B**;

**Table 2A:** Auxology during GH treatment for the young 3—9 years ITT population SDS versus the Swedish references (6, 49, 50).

Variables	Dose 33 µg (n=33)	Dose 67 µg (n=46)	p-value	Difference between groups Mean (95% CI)	Effect size
At GH start					
**Age (years)**	5.84 (1.48) 5.52 (3.04; 8.9) n=33	5.66 (1.76) 5.55 (3.07; 8.89) n=46	0.65	0.174 (−0.585; 0.921)	0.106
**Height_SDS_**	−2.78 (0.78) −2.84 (−3.97; −1.13) n=33	−2.78 (0.72) −2.71 (−5.39; −1.52) n=46	0.97	0.006 (−0.331; 0.344)	0.008
**Diff MPH_SDS_**	−2.51 (0.97) −2.35 (−4.23; −0.56) n=33	−2.70 (0.88) −2.7 (−4.5; −1.08) n=46	0.36	0.193 (−0.225; 0.614)	0.209
+ 1 yr All prepubertal					
**Mean GH dose_Year 1_** **(µg/kg/day)**	35.8 (5.8) 35.4 (25.5; 61) n=33	53.9 (7.6) 55.8 (28; 66.4) n=46	<.0001	−18.1 (−21.2; −15.0)	2.61
**Height_SDS_**	−2.28 (0.74) −2.26 (−3.57; −0.66) n=33	−1.93 (0.71) −1.9 (−4.35; −0.51) n=46	0.036	−0.350 (−0.675; −0.021)	0.486
At puberty start					
**Age (years)**	14.7 (1.1) 14.7 (12.5; 16.9) n=33	13.0 (1.4) 13.3 (9.1; 16.8) n=46	<.0001	1.65 (1.05; 2.25)	1.25
**Height_SDS_**	−1.10 (0.90) −1.09 (−3.18; 0.27) n=33	−0.42 (0.96) −0.40 (-3.02; 1.26) n=46	0.0017	−0.676 (−1.095; −0.247)	0.724
**Diff MPH_SDS_**	−0.83 (1.10) −0.60 (-3.39; 0.80) n=33	−0.34 (1.06) −0.37 (-2.41; 1.83) n=46	0.049	−0.489 (−0.971; −0.001)	0.455
**Mean GH dose_Pre puberty_ (µg/kg/day)**	34.8 (3.2) 34.1 (30.3; 46.7) n=33	56.8 (5.7) 58.1 (37.7; 65.4) n=46	<.0001	−22.0 (−24.2; −19.9)	4.55
**Gain in height_SDS_** **GH start - Puberty onset**	1.68 (0.67) 1.66 (0.48; 3.4) n=33	2.36 (0.74) 2.34 (0.81; 4.38) n=46	0.0002	−0.682 (−1.008; −0.363)	0.960

For continuous variables Mean (SD)/Median (Min; Max)/n= is presented.

For comparison between groups the Fisher´s Non Parametric PermutationTest was used for continuous variables.

The confidence interval for the mean difference between groups is based on Fishers non-parametric permutation test.

Effect size is absolute difference in mean/pooled SD.

GH, growth hormone; ns, not significant; SDS, standard deviation score; MPH, mid-parental height; DiffMPH, difference in SD score between the height of the girl and the heights of her parents; vrs: versus.

**Table 2B:** Auxology during GH treatment for the old ≥9 year ITT population versus the Swedish references (6, 49, 50).

Variables	Dose 33 µg (n=34)	Dose 67 µg (n=19)	p-value	Difference between groups Mean (95% CI)	Effect size
At GH start					
**Age (years)**	11.8 (2.1) 11.1 (9; 15.9) n=34	11.2 (1.5) 10.7 (9.2; 14.8) n=19	0.32	0.540 (−0.523; 1.652)	0.286
**Height SDS**	−2.80 (0.77) −2.76 (−4.43; −1.36) n=34	−2.82 (0.54) −2.93 (−3.96; −1.44) n=19	0.90	0.024 (−0.376; 0.426)	0.034
**Diff MPH_SDS_**	−2.57 (0.74) −2.49 (−3.95; −0.86) n=31	−2.90 (0.83) −2.86 (−4.01; −1.44) n=19	0.15	0.329 (−0.125; 0.788)	0.424
+ 1 yr All prepubertal					
**Average GH dose_Year 1_** **(µg/kg/day)**	33.7 (3.4) 33.7 (26.7; 40.7) n=34	56.5 (5.8) 56.1 (44; 71.4) n=19	<.0001	−22.7 (−25.2; −20.2)	5.21
**Height_SDS_**	−2.34 (0.77) −2.35 (−3.87; −0.81) n=28	−1.76 (0.60) −1.77 (−2.83; −0.41) n=19	0.0076	−0.582 (−1.002; −0.160)	0.825
At puberty start					
**Age (years)**	15.2 (1.5) 15.3 (11.2; 18) n=34	14.1 (1.7) 14.5 (11.2; 16.6) n=19	0.026	1.07 (0.13; 1.98)	0.668
**Height SDS**	−1.47 (0.72) −1.48 (−3.43; 0.18) n=34	−0.73 (0.71) −0.58 (−2.02; 0.30) n=19	0.0007	−0.732 (−1.145; −0.320)	1.02
**Diff MPH_SDS_**	−1.33 (0.84) −1.46 (−3.02; 0.67) n=31	−0.82 (0.81) −0.88 (−2.33; 0.68) n=19	0.040	−0.513 (−1.000; −0.026)	0.620
**Average GH dose** **prepuberty** **(µg/kg/day)**	34.0 (2.5) 33.4 (29.8; 41) n=32	58.2 (6.5) 59.1 (43.1; 68.9) n=19	<.0001	−24.2 (−26.8; −21.6)	5.49
**Gain in height_SDS_** **GH start-puberty onset**	1.33 (0.88) 1.38 (0; 3.43) n=34	2.09 (0.67) 2.16 (0.86; 3.06) n=19	0.0018	−0.756 (−1.219; −0.282)	0.928

For continuous variables Mean (SD)/median (Min; Max)/n= is presented.

For comparison between groups the Fisher’s non-parametric permutation test was used for continuous variables.

The confidence interval for the mean difference between groups is based on Fisher’s non-parametric permutation test.

Effect size is absolute difference in mean/pooled SD.

GH, growth hormone; ns, not significant; SDS, standard deviation score; MPH, mid-parental height; DiffMPH, difference in SD score between the height of the girl and the heights of her parents; vrs, versus.

**Table 3A:** Auxology at adult height for the young 3—9 years ITT population versus the Swedish references (6, 49, 50).

Variables	Dose 33 µg (n=33)	Dose 67 µg (n=46)	p-value	Difference between groups Mean (95% CI)	Effect Size
At adult height					
**Age (years)**	17.3 (1.2) 17 (15.6; 21.4) n=33	16.1 (1.2) 16 (13.4; 19.8) n=46	0.0002	1.13 (0.60; 1.66)	0.968
**Adult height (cm)**	153.7 (5.4) 155.1 (143.7; 165.4) n=33	157.2 (5.8) 156.8 (145.1; 168.3) n=46	0.0083	−3.47 (−6.02; −0.90)	0.617
**Height_SDS_**	−2.28 (0.89) −2.05 (−3.93; −0.36) n=33	−1.71 (0.95) −1.77 (−3.7; 0.12) n=46	0.0083	−0.571 (−0.990; −0.148)	0.617
**Diff MPH_SDS_**	−2.01 (0.99) −1.84 (−4.53; −0.43) n=33	−1.63 (0.93) −1.66 (−4.07; 0.49) n=46	0.083	−0.384 (−0.816; 0.050)	0.403
**Gain in height_SDS_** **Puberty onset** − **adult height**	−1.18 (0.38) −1.21 (−1.88; −0.37) n=33	−1.29 (0.56) −1.39 (−2.63; 0) n=46	0.36	0.105 (−0.122; 0.331)	0.213
**Gain height (cm)** **Puberty onset—adult height**	3.25 (2.63) 2.7 (−0.2; 10.1) n=33	7.67 (5.60) 6.2 (0; 23.3) n=46	<.0001	−4.42 (−6.50; −2.33)	0.958
**Mean GH dose_Total_** **(µg/kg/day)**	34.9 (3.5) 34 (30.3; 48.1) n=33	55.1 (6.2) 55.6 (37.3; 65.9) n=46	<.0001	−20.1 (−22.5; −17.8)	3.83
**Time on GH (years)**	10.2 (1.8) 10.3 (5.3; 13) n=33	9.43 (2.02) 9.72 (5.36; 13.87) n=46	0.082	0.776 (−0.098; 1.670)	0.400
**Duration puberty (years)**	2.57 (1.05) 2.4 (0.76; 5.28) n=33	3.09 (1.59) 3.08 (0; 9.26) n=46	0.11	−0.517 (−1.143; 0.113)	0.373
**Gain in height_SDS_** **GH start − adult height**	0.49 (0.68) 0.53 (-0.69; 1.69) n=33	1.07 (0.73) 1.00 (−0.52; 3.27) n=46	0.0006	−0.577 (−0.905; −0.257)	0.816

For continuous variables, mean (SD)/median (Min; Max)/n= is presented.

For comparison between groups the Fisher’s non-parametric permutation test was used for continuous variables.

The confidence interval for the mean difference between groups is based on Fisher’s non-parametric permutation test.

Effect size is absolute difference in mean/pooled SD.

AH, adult height; GH, growth hormone; SDS, standard deviation score; MPH, mid-parental height; DiffMPH, difference in SD score between the height of the girl and the heights of her parents.

**Table 3B:** Auxology at adult height for the ≥9 year old ITT population versus the Swedish population (6, 49, 50).

Variables	Dose 33 µg (n=34)	Dose 67 µg (n=19)	p-value	Difference between groups Mean (95% CI)	Effect Size
At adult height					
**Age (years)**	18.5 (2.0) 18.2 (15.9; 25.9) n=34	17.2 (1.2) 17.6 (14.8; 18.9) n=19	0.0050	1.29 (0.36; 2.32)	0.741
**Adult height (cm)**	156.5 (5.0) 156.2 (144; 169.2) n=34	159.9 (3.8) 159.7 (150.4; 167) n=19	0.015	−3.32 (−5.99; −0.68)	0.717
**HeightSDS_t)_**	−1.82 (0.83) −1.87 (−3.88; 0.27) n=34	−1.27 (0.62) −1.3 (−2.83; −0.1) n=19	0.015	−0.546 (−0.985; −0.112)	0.717
**Diff MPH_SDS_**	−1.67 (0.94) −1.84 (−3.92; 0.03) n=31	−1.35 (0.78) −1.44 (−2.71; −0.18) n=19	0.23	−0.317 (−0.842; 0.193)	0.358
**Gain in height SDS** **Puberty onset—adult height**	−0.35 (0.63) −0.42 (−1.78; 1.63) n=34	−0.54 (0.54) −0.69 (−1.33; 0.66) n=19	0.29	0.186 (−0.147; 0.535)	0.311
**Gain in height (cm)** **Puberty onset—adult height**	7.15 (5.49) 5.7 (0.9; 21.1) n=34	9.16 (6.77) 5.6 (1.4; 26.8) n=19	0.25	−2.01 (−5.45; 1.52)	0.337
**Mean GH dose_Total_** **(µg/kg/day)**	34.6 (3.6) 33.2 (30.4; 46.1) n=34	57.7 (6.7) 59.1 (41.9; 69.1) n=19	<.0001	−23.1 (−26.0; −20.3)	4.67
**Time on GH (years)**	5.19 (1.69) 5.55 (1.99; 8.29) n=34	5.28 (1.15) 5.37 (2.62; 7.14) n=19	0.85	−0.089 (−0.961; 0.774)	0.059
**Duration puberty (years)**	3.30 (1.87) 3.33 (0.61; 8.7) n=34	3.07 (1.04) 2.77 (1.61; 5.37) n=19	0.65	0.225 (−0.689; 1.178)	0.139
**Gain in height_SDS_** **GH start—adult height**	0.98 (0.89) 1.04 (−1.32; 2.66) n=34	1.55 (0.55) 1.61 (−0.19; 2.23) n=19	0.012	−0.570 (−1.031; −0.125)	0.721

For continuous variables Mean (SD)/Median (Min; Max)/n= is presented.

For comparison between groups the Fisher’s non-parametric permutation test was used for continuous variables.

The confidence interval for the mean difference between groups is based on Fishers non-parametric permutation test.

Effect size is absolute difference in mean/pooled SD.

AH, adult height; GH, growth hormone; SDS, standard deviation score; MPH, mid-parental height; DiffMPH, difference in SD score between the height of the girl and the heights of her parents.

B: for comparisons for all girls versus dose, see **Supplementary Tables S3A–C**, and

C: for comparisons for all girls versus age, **Supplementary Tables 4A–C**, all versus the Swedish reference for non-TS girls (6).

Corresponding information for the PP population is presented in **Supplementary Tables S1, S2, S6, S7**.

Auxological data according to a TS reference (5) is presented in **Table 4**, for the ITT population and in **Supplementary Table S5** for the PP population.

**Table 4:** Growth from birth to adult height in the ITT study subgroups; expressed in SDS versus the Turner reference (5).

	Group									
	GH start 3- <9					GH start 9-				
Variables	Dose 33 µg	Dose 67 µg	p-value	Difference between groups Mean (95% CI)	Effect Size	Dose 33 µg	Dose 67 µg	p-value	Difference between groups Mean (95% CI)	Effect Size
	n=33	n=46				n=34	n=19			
At birth										
**Height SDS**	−0.58 (1.08) −0.24 (−3.04; 0.96) n=33	−0.21 (0.95) −0.24 (−2.24; 1.76) n=46	0.12	−0.365 (−0.824; 0.089)	0.364	−0.01 (0.86) 0.16 (−2.24; 1.56) n=34	−0.24 (1.11) −0.16 (−2.24; 0.96) n=18	0.41	0.235 (−0.333; 0.785)	0.248
At GH start										
**Height SDS**	−0.24 (0.88) −0.30 (−1.55; 1.49) n=33	−0.26 (0.86) −0.25 (−3.15; 1.45) n=46	0.94	0.015 (−0.378; 0.407)	0.017	0.21 (0.96) 0.14 (−1.81; 2.02) n=34	0.13 (0.67) −0.01 (−0.95; 1.93) n=19	0.78	0.071 (−0.425; 0.573)	0.082
+ 1 year all prepubertal										
**Height_SDS_**	0.37 (0.80) 0.32 (−0.98; 2.16) n=32	0.81 (0.86) 0.89 (−1.85; 2.74) n=46	0.026	−0.441 (−0.829; −0.056)	0.528	0.82 (0.88) 0.77 (−1.06; 2.61) n=27	1.43 (0.70) 1.43 (0.39; 3.05) n=19	0.016	−0.605 (−1.084; −0.121)	0.748
At puberty start										
**Height SDS**	2.30 (0.97) 2.33 (0.17; 3.79) n=33	3.05 (1.07) 3.1 (−0.1; 4.92) n=46	0.0016	−0.753 (−1.219; −0.282)	0.730	1.90 (0.75) 1.88 (−0.02; 3.64) n=34	2.64 (0.82) 2.86 (1.07; 3.83) n=19	0.0018	−0.742 (−1.196; −0.292)	0.952
At adult height										
**Height SDS**	1.85 (1.01) 1.83 (−0.07; 3.62) n=33	2.81 (1.01) 2.9 (0.77; 4.86) n=46	<.0001	−0.962 (−1.419; −0.503)	0.952	2.04 (0.80) 2.2 (−0.45; 3.74) n=34	2.87 (0.50) 2.97 (1.78; 3.85) n=19	0.0002	−0.822 (−1.234; −0.427)	1.16
Height gain										
**Gain in height_SDS_** **GH start—Puberty onset**	2.54 (0.69) 2.42 (1.54; 4.48) n=33	3.31 (0.78) 3.34 (1.74; 5.3) n=46	<.0001	−0.767 (−1.105; −0.429)	1.03	1.70 (1.08) 1.78 (0; 4.06) n=34	2.51 (0.82) 2.64 (1.08; 3.69) n=19	0.0066	−0.812 (−1.381; −0.233)	0.815
**Gain in height_SDS_** **Puberty onset—adult height**	−0.45 (0.28) −0.43 (−1.20; −0.01) n=33	−0.24 (0.50) −0.27 (−1.18; 0.92) n=46	0.032	−0.209 (−0.401; −0.020)	0.499	0.14 (0.49) 0.04 (−0.59; 1.72) n=34	0.22 (0.66) 0.16 (−0.85; 2.17) n=19	0.61	−0.080 (−0.397; 0.246)	0.143
**Gain in height_SDS_** **GH start—adult height**	2.09 (0.72) 2.07 (0.69; 3.44) n=33	3.07 (0.76) 3.01 (1.47; 5.08) n=46	<.0001	−0.977 (−1.321; −0.633)	1.31	1.84 (1.03) 1.99 (−0.07; 3.99) n=34	2.73 (0.62) 2.91 (1.41; 3.63) n=19	0.0010	−0.892 (−1.416; −0.372)	0.983

For continuous variables, Mean(SD)/Median (Min; Max)/n= is presented.

For comparison between groups, the Fisher’s non-parametric permutation test was used for continuous variables.

The confidence interval for the mean difference between groups is based on Fisher’s non-parametric permutation test.

Effect size is absolute difference in mean/pooled SD.

GH, growth hormone; SDS, standard deviation score.

**Figure 2** show changes in height_SDS_ versus both references.

#### 4.1.1 Prepubertal gain in height_SDS_

*The young age group (3–9 years).* At the start of treatment, there was no difference in mean height_SDS_ (both –2.8) or mean age (5.8 versus 5.7 years, respectively) between the low- and high-dose groups. At 1 year of treatment (see **Table 2A**), and at 2 years of treatment, the GH_33_ group was significantly shorter than the GH_67_ group, mean (SD) of –2.04 (0.77) versus –1.48 (0.77), respectively, a difference in height_SDS_ of 0.56 (p < 0.01) increased to 0.96 (p<0.0001) after 4 years on treatment and was maintained throughout the childhood growth period. Only two girls in the GH_67_ group started Oxandrolone during the first year of GH treatment, and no further girls started this treatment during the second year.

At start of puberty, the GH_33young_ group had reached a height_SDS_ of −1.10 compared with −0.423 for the GH_67young_ group (p=0.0017); mean duration of prepubertal treatment was 8.9 and 7.3 years, respectively. Total prepubertal gain in height_SDS_ from GH start to last prepubertal visit/start of puberty was 1.68 for the GH_33young_ group and 2.36 for the GH_67young_ group (p=0.0002); the mean difference in height_SDS_ was −0.68 (95% CI, −1.008 to −0.363) (**Table 2A**, **Figure 3**).

*The old age group (>9 years).* At start of treatment, there was no difference in mean height_SDS_ (both −2.8) or mean age (11.8 versus 11.2 years, respectively) between the low- and high-dose groups. At 1 year on GH treatment, height_SDS_ was −2.34 for the GH_33old_ group and −1.76 for the GH_67old_ group (p=0.0076). During the first year of treatment, 13 (68%) girls in the GH_67_ group were started on Oxandrolone, while 9 (26%) from the GH_33_ group were started on Oxandrolone during the second year (**Table 2B**, **Figure 3**).

At start of puberty, height_SDS_ was −1.47 for the GH_33old_ group versus −0.734 for the GH_67old_ group (p=0.0007). After 3.4 and 2.9 years of GH treatment, respectively, total prepubertal gain in height_SDS_ was 1.33 for the GH_33old_ group versus 2.09 for the GH_67old_ group (p=0.0018); the mean difference between groups was 0.756 (95% CI, −1.219 to −0.282; **Table 2B**, **Figure 3**).

*Comparisons of GH dose between the age groups.* At +1 year on GH 33 µg/kg/day, there was no significant difference in mean dose between the young and old groups: GH_young_ and GH_old_ groups received 35.8 and 33.7 µg/kg/day, respectively (p=0.077); at the last prepubertal visit, doses were 34.8 and 34.0 µg/kg/day, respectively (p=0.31).

For the dose group 67 µg/kg/day, there were also no significant differences in terms of dose between the young and old groups at either time point (comparable values for +1 year were 53.9 and 56.5 µg/kg/day, respectively, and that at last prepubertal visit were 56.8 and 56.2 µg/kg/day, respectively(p=0.39)) (**Tables 2A, B**).

#### 4.1.2 Pubertal growth

*The young age group (3–9 years).* A negative change in height_SDS_ from start of puberty to AH was found: –1.18 for the GH_33_ versus –1.29 for the GH_67_ group (p=0.36). When expressed in centimeters, the corresponding values for pubertal growth were 3.25 and 7.67 cm, respectively (p<0.0001); the mean difference between groups was −4.42 (95% CI, −6.50 to −2.33) (**Table 3A**, **Figure 3**).

*The old age group (>9 years).* The change in height_SDS_ during puberty for this group was also negative: by −0.351 for the GH_33_ group versus −0.536 for the GH_67_ group (p=0.29); mean difference was 0.15 (95% −0.147 to 0.535). When expressed in centimeters, height gain during puberty was 7.15 and 9.16 cm for the GH_33_ and the GH_67_ group (p=0.25), respectively (**Table 3B**, **Figure 3**).

*Comparisons of GH dose between the age groups.* The calculation of mean dose for the total treatment period was 34.9 vs. 34.6 µg/kg/day for the young and old GH_33_ groups, respectively. The corresponding values for the young and old GH_67_ groups were 55.1 vs. 57.7 µg/kg/day.

#### 4.1.3 Total gain in height_SDS_

*The young age group (3–9 years).* For the young group of girls, the total gain in height_SDS_ was 0.49 for the GH_33_ group compared with 1.07 for the GH_67_ group (p=0.0006); mean difference was 0.57 (95% CI, −0.905 to −0.257). Total time on GH treatment was 10.2 versus 9.43 years, respectively (p=0.082) (**Table 3A**, **Figure 3**).

*The old age group (>9 years).* For the old group, the total gain in height_SDS_ was 0.98 for the GH_33_ group versus 1.55 for the GH_67_ group (p=0.012); mean difference was 0.57 (95% CI, −1.031 to −0.125). Total time on GH treatment was 5.19 and 5.28 years, respectively (p=0.85) (**Table 3B**, **Figure 3**).

*Comparisons between and over age groups.* When the total gain in height_SDS_ from GH start to AH in the PP population was compared for the two dose groups, independent of age at GH start, the GH_33_ groups experienced a significantly lower gain in height_SDS_ (0.893) compared to the GH_67_ groups (1.23) (p=0.020); mean difference was 0.391 (95% CI, −0.714 to −0.064) (**Supplementary Table S5**).

#### 4.1.4 Adult height

*The young age group (3–9 years).* Adult height_SDS_ was −2.28 for the GH_33young_ versus −1.71 for the GH_67young_ group (p=0.0083); mean difference was 0.57 (95% CI, −0.990 to −0.148). When expressed in centimeters, AH was 153.7 versus 157.2 cm in the low- and high-dose groups, respectively (p=0.0083), mean difference was 3.5 cm (95% CI, −6.02 to −0.90) (**Table 3A**, **Figure 4**).

*The old age group (>9 years).* Adult height_SDS_ was −1.82 for the GH_33_ versus −1.27 for the GH_67_ group (p=0.015); mean difference was 0.546 (95% CI, −0.985 to −0.112). When expressed in centimeters, AH was 156.5 versus 159.9 cm in the low- and high-dose groups, respectively (p=0.015); mean difference was 3.4 cm (95% CI, −5.99 to −0.68) (**Table 3B**, **Figure 4**).

*Comparisons between and over age groups*. If considering only age at GH start and not dose in the PP population, the GH_young_ group attained an AH_SDS_ of −1.95, while the GH_old_ group attained an AH_SDS_ of −1.60 (p=0.075); mean difference was 0.341 (95% CI, −0.709 to 0.037) (**Supplementary Table S6C**).

#### 4.1.5 Spontaneous or induced puberty

*Data from the girls with spontaneous puberty.* Of the 32 girls with spontaneous onset of puberty in the ITT population, 22 attained full pubertal development spontaneously. Data from these 22 girls were as follows: the groups GH33/67_young_ and GH 33/67_old_ reached AH_SDS_ mean (SD) of −2.32(0.47)/−2.13(0.85) versus −2.00(0.89)/−1.84(0.54), respectively, with pubertal height gain (cm) 2.4/12.1 versus 9.7/17.6. Pubertal duration ranged from 1.8 to 4.2 years.

*Data from the girls with induced puberty*. Most girls needed puberty induction; divided into the groups GH33/67_young_ and GH 33/67_old_, AH_SDS_ was mean (SD) of −2.28(0.92)/−1.59(0.95) versus −1.72(0.80)/−1.01(0.47), respectively.

### 4.2 Age outcomes

#### 4.2.1 Age at puberty onset

##### 4.2.1.1 Induced puberty

*The young age group, 3–9 years.* Age at start of puberty was significantly greater for the GH_33young_ compared with the GH_67young_ group (14.7 versus 13.0 years, respectively) (p<0.0001) and occurred at a height_SDS_ of −1.10 versus −0.423, respectively (p=0.0017) (**Table 2A**). Age at pubertal onset for those started on ERT was significantly greater for the GH_33young_ compared with the GH_67young_ group (14.6 versus 13.5 years, respectively) (p<0.001).

*The old age group, >9 years.* Girls in the older age group receiving GH_33_ were older at the start of puberty than those receiving GH_67_ (15.2 vs. 14.1 years, respectively) (p=0.026). Height_SDS_ at start of puberty was −1.47 in the low versus −0.734 in the high-dose group (p=0.0007) (**Table 2B**). However, age at induction of puberty was similar for low- and high-dose groups (15.5 versus 15.2 years, respectively).

*Comparisons between and over age groups*. When age at onset of puberty was compared for the four subgroups, puberty was induced at a significantly earlier age for those in the GH_67young_ group versus the other groups (**Figure 4**). For those girls receiving oral EE2, age at onset of puberty ranged from mean (SD) 13.8 (1.1) to 15.5 (1.1) years, with lower age in the younger group. For those receiving transdermal estradiol patches, mean age at onset of puberty was 13.4 (1.1)–16.8 (n=1) years, with lowest age in the GH_67young_ group.

##### 4.2.1.2 Spontaneous puberty

Spontaneous onset of puberty was seen in 32 girls, 10 of whom later needed ERT (GH_young33_: n=1; GH_young67_: n=2; GH_old33_: n=6; and GH_old67_: n=1).

*The young age group, 3–9 years.* Age at onset of spontaneous puberty was significantly greater for the GH_33_ compared with the GH_67_ group [mean (SD) 16.1 (0.2) years (n=2) versus 11.7 (1.4) years (n=12)] (p<0.05).

*The old age group, >9 years.* For the older girls, those on GH_33_ also started puberty later, at a mean (SD) age of 14.6 (1.9) years (n=12), compared with those on GH_67_, who started puberty at 11.8 (0.5) years (n=6) (p<0.01).

#### 4.2.2 Age at adult height

*The young age group: 3–9 years.* The GH_33young_ group reached AH significantly later, at age 17.3 years, compared with GH_67young_ group, who reached AH at 16.1 years (p=0.0002), with a duration of puberty of 2.57 versus 3.09 years (p=0.11), respectively (**Table 3A**).

*The old age group, >9 years.* The older girls on GH_33_ reached AH at 18.5 years compared to 17.2 years for the GH_67_ group (p=0.0050), with a duration of puberty of 3.30 and 3.07 years (p=0.65), respectively (**Table 3B**).

##### 4.2.2.1 Comparisons between and over age groups

When age at AH was compared between the two dose groups in the PP population, independent of age at GH start, the GH_33_ group was significantly older than the GH_67_ group when they attained AH (17.8 versus 16.6 years, respectively; p<0.0001) (**Supplementary Table S6C**). When the total gain in height_SDS_ from GH start to AH was compared for the two dose groups, independent of age, the GH_33_groups gained significantly less height_SDS_ (0.839) compared with the GH_67_ groups (1.23), (p=0.020) (**Supplementary Table S6C**).

When age at AH was compared between the groups starting treatment when young versus old in the PP population, the GH_young_ reached AH at a mean age of 16.8 years, while the corresponding age for the GH_old_ was 17.9 years (p=0.0002) (**Supplementary Table S7C**).

All analyses made in the ITT population were also performed in the PP population and presented in **Supplementary Tables S1, S2, S5–S7**.

### 4.3 Multivariable linear regression analyses

In this study, we selected as height outcomes gain in height_SDS_ and attained AH in both SDS and centimeters, and as age outcomes, age at onset of puberty, and age at attained AH. Stepwise forward regression models were used to explain the variation in these outcomes using independent available variables at two time points of interest, at GH start, and at onset of puberty. The age of the girls with TS at GH start was the only common independent variable in all models, followed by the selected GH dose in the models that explain variation in height gain and the ages at pubertal onset and AH but not for AH. For the height outcomes, the parental heights, *per se* or as the difference to the height of the girl, were selected for as an informative variable. No major differences were found when using the two populations, either the entire ITT group of 132 girls or only those in the PP population of 89 girls, who followed the protocol. See **Table 5**, for the ITT population and **Table 6** for the PP population.

**Table 5:** Multivariable linear regression analyses, ITT population 132 girls with TS.

		Independent variables Beta/(95% CI)/p-value								
		Before GHstart	At GHstart			1st year	Prepub period	At Pubertal onset		
		MPH (SDS)	Age (years)	Height (SDS)	Diff MPH (SDS)	Mean GH dose (µg/kg/day)	Gain in height(SDS)	Age (years)	Height (SDS)	Diff MPH (SDS)
Dependent	R-square									
Gain in height										
Prepub gain SDS @GH	0.2521		−0.05 (−0.09; −0.01) p=0.012	−0.26 (−0.44; −0.08) p=0.0049		0.02 (0.01;0.04) p<0.0001				
Pub gain SDS @puberty	0.5278		0.11 (0.09;0.14) p<0.0001							−0.21 (−0.29; −0.12) p<0.0001
Total gain SDS @GH	0.2714		0.06 (0.03;0.10) p=0.0008	−0.21 (−0.39; −0.03) p=0.024	−0.23 (−0.38; −0.08) p=0.0034	0.02 (0.01;0.03) p=0.0015				
Total gain SDS @puberty	0.6916		0.11 (0.08;0.13) p<0.0001		−0.14 (−0.24; −0.05) p=0.0032		0.74 (0.64;0.85) p<0.0001			
Adult height										
Adult height SDS @GH	0.3966	0.28 (0.12;0.43) p=0.0005	0.05 (0.02;0.09) p=0.0059	0.51 (0.31;0.70) p<0.0001						
Adult height cm @GH	0.3966	1.68 (0.75;2.61) p=0.0005	0.32 (0.09;0.55) p=0.0059	3.07 (1.86;4.28) p<0.0001						
Adult height SDS @puberty	0.7471	0.17 (0.07;0.26) p=0.0008	0.11 (0.09;0.14) p<0.0001						0.75 (0.65;0.85) p<0.0001	
Adult height cm @puberty	0.7471		0.68 (0.53;0.84) p<0.0001						5.58 (4.94;6.23) p<0.0001	−1.01 (−1.59; −0.43) p=0.0008
Age at Pubertal onset										
Age Puberty @GH	0.2258		0.18 (0.10;0.26) p<0.0001			−0.04 (−0.06; −0.01) p=0.0022				
Age at Adult height										
Age Adult height @GH	0.2999		0.23 (0.15;0.30) p<0.0001			−0.03 (−0.05; −0.01) p=0.0032				
Age Adult height @puberty	0.5097		0.13 (0.06;0.20) p=0.0002					0.42 (0.28;0.56) p<0.0001	−0.49 (−0.72; −0.26) p<0.0001	

GH, growth hormone; SDS, standard deviation score; MPH, mid-parental height; DiffMPH, difference in SDS between the height of the girl and the heights of her parents. SDS calculated versus the Swedish population for the girls (6, 57) and for the parents (58). ITT, intention to treat population; PP, per protocol population; @GHstart, model with available independent variables at GHstart; @puberty, models with available independent variables at pubertal onset.

**Table 6:** Multivariable linear regression analyses, PP population, 89 girls with Turner syndrome.

		Independent variables Beta/(95% CI)/p-value										
		Before GHstart				At GHstart			1st year	Prepub period	At Pubertal onset	
		Karyotype	MPH (SDS)	Mother height (SDS)	Father height (SDS)	Age (years)	Height (SDS)	Diff MPH (SDS)	Mean GHdose (µg/kg/day)	Gain in height (SDS)	Age (years)	Height (SDS)
Dependent	R-square											
Gain in Height												
Prepub gain SDS @GH	0.4212			0.25 (0.11;0.40) p=0.0006		−0.05 (−0.09; −0.01) p=0.026	−0.45 (−0.64; −0.27) p<0.0001		0.02 (0.01;0.03) p=0.0003			
Pub gain SDS @puberty	0.4747					0.13 (0.10;0.16) p<0.0001						
Total gain SDS @GH	0.4131	−1.17 (−1.91; −0.42) p=0.0024				0.07 (0.03;0.11) p=0.0007	−0.24 (−0.43; −0.04) p=0.017	−0.28 (−0.44; −0.11) p=0.0013	0.02 (0.01;0.03) p=0.0050			
Total gain SDS @puberty	0.7071					0.12 (0.09;0.15) p<0.0001				0.83 (0.71;0.95) p<0.0001		
Adult height												
Adult height SDS @GH	0.4245		0.28 (0.11;0.46) p=0.0019			0.07 (0.02;0.11) p=0.0034	0.45 (0.22;0.67) p=0.0002					
Adult height cm @GH	0.4245		1.73 (0.66;2.80) p=0.0019			0.41 (0.14;0.67) p=0.0034	2.72 (1.35;4.10) p=0.0002					
Adult height SDS @puberty	0.7726				0.10 (0.00;0.20) p=0.047	0.12 (0.09;0.14) p<0.0001						0.83 (0.72;0.95) p<0.0001
Adult height cm @puberty	0.7726				0.60 (0.01;1.19) p=0.047	0.71 (0.53;0.88) p<0.0001						5.06 (4.37;5.75) p<0.0001
Age at Pubertal onset												
Age Puberty @GH	0.2841					0.17 (0.08;0.25) p=0.0004			−0.05 (−0.08; −0.03) p=0.0002			
Age at adult height												
Age Adult height @GH	0.3244					0.18 (0.10;0.26) p<0.0001			−0.04 (−0.07; −0.02) p=0.0004			
Age adult height @puberty	0.4476					0.10 (0.02;0.18) p=0.019				−0.46 (−0.77; −0.14) p=0.0046	0.42 (0.26;0.58) p<0.0001	

GH, growth hormone; SDS, standard deviation score; MPH, mid-parental height; DiffMPH, difference in SDS between the height of the girl and the heights of her parents. SDS calculated versus the Swedish population for the girls (6, 57) and for the parents (58). ITT, intention to treat population; PP, per protocol population; @GHstart, model with available independent variables at GHstart; @puberty, models with available independent variables at pubertal onset.

#### 4.3.1 Height outcomes

##### 4.3.1.1 Gain in height SDS

The prepubertal gain was to 25% (ITT) and to 42% (PP) explained by three variables at GH start: age, height, and the selected GH dose for ITT, with mother height added for PP population.

The pubertal gain was to 53% (ITT) and to 48% (PP) explained by two variables at pubertal onset: age at GH start and diffSDS.

The total gain was to 27% (ITT) and to 41% (PP) explained at GH start for ITT with age, height, and diffSDS at GH start, and at pubertal onset to 69% with also prepubertal gain.

PP at GH start was to 41%, with age, diffSDS, and chromosomes (the only model), and at pubertal onset to 71%, using age and height at GH start with the selected GH dose and prepubertal height gain.

##### 4.3.1.2 Adult height_SDS_

At GH start, the variation was explained to 40% (ITT) and to 43% (PP) and explained by three available variables, namely, age, height, and diffSDS (only ITT).

At pubertal onset, the variation could be explained to 75% (ITT) and to 77% (PP) by age at GH start, diffSDS [and for PP addition of parental heights (MPH, mother height or father height)], and height at pubertal onset.

#### 4.3.2 Age outcomes

##### 4.3.2.1 Age at pubertal onset

Age at pubertal onset could at GH start be explained to 23% (ITT) and to 28% (PP) by age and the selected GH dose.

##### 4.3.2.2 Age at adult height, AH

Age at AH was to 30% (ITT) and to 32% (PP) explained by age at GH start and the selected GH dose, and at pubertal onset to 51% (ITT) and to 45% (PP) by age at GH start and age and height at pubertal onset (ITT).

### 4.4 Safety

Oxandrolone was started at dose 0.05 mg/kg/day with cautious monitoring according to voice deepening and other androgen effects. Dose reduction was allowed by the investigator, no girl treated with oxandrolone received a daily dose lower than 0.025 mg/kg. No girl was diagnosed with diabetes mellitus. If signs of celiac disease or thyroid disturbance became evident, proper treatment was instantly instituted and occurred after enrollment in two girls. FSH and LH were followed yearly from age 7 years (or at diagnose) for early diagnose of possible gonadal failure. A total of 10 girls with spontaneous onset of puberty needed supportive estrogen substitution.

## 5 Discussion

### 5.1 Principal findings

The major finding from this study based on a temporal design of multicentre clinical trials was that a normal adult height can be attained for girls with TS when treated with rhGH prior to puberty, irrespective of age when the diagnosis of TS was made, from early childhood through to ~16 years of age. This can be achieved by individualized treatment with GH (and possibly oxandrolone for growth support) with doses adapted according to individual GH responsiveness, as revealed by the first-year growth response. Pubertal growth and development can also be optimized using the addition of a combination of ERT and, where needed, androgens. Transdermal 17β-estradiol is preferred as possible to mimic physiology regarding onset, progress, and duration.

A GH-dose effect on growth was found in both early and late diagnosed girls relative to puberty, even though the between-group difference in mean GH dose was narrower than intended, 35 versus 57 µg/kg/day, respectively, with broad ranges indicating dose individualization. Total height gain was lowest for the young age group on the low GH dose (0.5 SDS) and greatest for the old age group on the high GH dose (1.55 SDS). A substantial prepubertal gain in height was achieved. However, this relative height gain was partly lost during puberty through subnormal pubertal growth. Although these findings suggest that GH dose is a key factor, later age at diagnose is known to be associated with more subtle features of TS, and therefore, comparisons within each age group will be the most relevant. AH was within acceptable ranges for all four groups, since the great gain achieved before puberty overcame the subnormal pubertal growth. Mean AH for all groups were close to or within the reference range for the normal population; height_SDS_ was –2.28 for the group with the lowest mean AH of 153.7 cm (GH_young33_) and –1.27 for the group with the greatest mean AH of 159.9 cm (GH_old67_). It is of note that although girls who started treatment later with the lower GH dose did attain a mean AH_SDS_ within normal range (–1.82; 156.5 cm), this was only achieved when they were 18.5 years of age. In comparison, AH was attained at the age of 17.2 years in the GH_67old_ group. Irrespective of which GH dose they received, most girls in the old age group had adjuvant oxandrolone treatment from the first or second year on GH treatment before start of ERT. Thus, as shown in a previous study, GH treatment may increase AH, although delayed as in the present study, even when started late relative to the onset of puberty (31). Both pubertal duration and gain in height was low for all groups, resulting in that the prepubertal gain was partly lost during puberty. Thus, with improved treatment during puberty, initially using physiologically low transdermal 17β-estradiol available nowadays (38, 57, 59), the intentional delay in ERT and age for AH in these trials might not have been necessary (58).

### 5.2 GH treatment for growth

#### 5.2.1 GH secretion and GH dose

In the late 1980s when the Swedish trials were planned and initiated, rhGH was only approved for use in children with GHD, and very little was known about the effects of GH dose on height gain. At this time, the parallel study by the group of Hintz and Rosenfeld that led to the approval of GH treatment (50 µg/kg/day) for TS were ongoing (60, 61). During the years to come, the effect of GH dose on girls with TS was studied in several trials in many countries (10, 22–31) and in international outcome databases (32–36). Results have convincingly shown the importance of GH dose during prepuberty; low doses are not enough (26, 28, 62, 63), while higher doses are (29). The present Swedish trial results strongly support these findings.

The need for a different GH dose in girls with TS compared with children with GHD is today not surprising. The GH dose approved for treatment of GHD was estimated based on the GH secretion rate of healthy children (64). However, compared with non-TS girls, girls with TS have a different GH secretory pattern (51) due to that they mainly secrete the 20-kDa isoform rather than the normally more abundant 22 kDa isoform (65, 66). The 20-kDa isoform has a longer half-life and is associated with greater metabolic and less longitudinal growth-promoting effects (65, 66). The longer half-life of the 20 kDa form will also result in higher GH trough levels, resulting in constant serum GH concentration, something known to be negative for growth (67). Furthermore, it is known that administration of exogenous rhGH (the 22-kDa isoform) reduces endogenous GH secretion (of any isoform) for hours, owing to the well-known negative feedback mechanism by GH on its own secretion, with the duration of the reduction depending on the amount and depth of the injection (56).

In our studies, the intended GH doses were not adhered to by many of the investigators: the low dose, which was intended to be 33 µg/kg/day, instead became on average 37 µg/kg/day, whereas the high dose of 67 µg/kg/day became on average 57 µg/kg/day, thereby reducing the study dose–response range. Thus, the GH doses in at least a quarter of the 132 girls with TS were actually individualized, which resulted in the exclusion of data from these girls from the PP analysis. This high degree of deviation from protocol is of note, as it may indicate that the range of responsiveness was narrower than expected and that signs of overdose, such as water retention or the development of acromegalic features, may have occurred at a lower-than-expected dose in this patient group.

#### 5.2.2 GH response and GH responsiveness

Growth response varies considerably, even between individuals with the same diagnosis and general characteristics who have received comparable GH doses. In this context, it may be helpful to imagine that, for each individual child, there is a set point of balance between GH secretion and GH responsiveness (68), and that this balance differs during the different growth phases (68, 69) and between tissues. We know, for example, that the bone tissue seems to be the least sensitive tissue in the body to GH, whereas the brain is the most sensitive (70, 71).

Responsiveness to GH can be estimated using the first year growth response to a specific GH dose (15) or using a prediction model for estimation of growth response (16, 17, 72). According to the KIGS TS prediction model (18), which estimates first year prepubertal GH growth response in centimeters, the most important variable was GH dose (studied dose range, 23–52 µg/kg/day) followed by age, weight, and oxandrolone treatment. This is consistent with the present findings. The fact that the model showed that the observed first year growth response explained most of the following second year growth response highlights the importance of considering individual GH responsiveness. In the present study, by multivariable regression model analysis, we identified GH dose to be an important variable to explain the variation in prepubertal gain in height_SDS_: belonging to the high-dose group was associated with a greater gain. Additionally, being young at GH start was associated with a greater growth response. Together, these data suggest that the growth deficit associated with a late GH start can, at least partly, be compensated for by a higher GH dose during the remaining or extended prepubertal growth period. The association of young age at GH start and thereby greater growth response/responsiveness has previously been reported in girls with TS (18, 73), as in other diagnostic groups including children with GHD and ISS (16, 74, 75). When calculating growth response versus the TS growth reference, GH dose *per se* was, together with young age, found to explain most of the variation in total growth response, indicating the individual GH responsiveness (32). Young age at GH start also prevents short stature already from childhood by allowing more prepubertal years for growth (30, 32, 73).

### 5.3 Estrogen replacement therapy for growth and pubertal development

This analysis showed height gain during puberty to be low or absent, with many girls experiencing a loss in relative height during this period despite growing well when receiving GH treatment prior to puberty. As per the protocol, ERT was used to initiate puberty at an appropriate time from 13 years onwards as determined jointly by the physician and girl; data showed that puberty was initiated between 13 and 15.2 years of age, and that in all groups, girls were of an average height close to 150 cm at the time. This later than normal start of ERT may be due to prior clinical experience of little further growth after the start of estrogen therapy with EE2 at the doses used (33). Even though the EE2 dose used at that time was estimated to be low, it could still have had a higher-than-expected estrogen effect resulting in growth plate maturation being too rapid, leaving too little time for pubertal growth. Consistent with this, the mean duration of puberty was only approximately 3 years (2.6–3.3 years). Breast development also suffers when ERT dose increments are too rapid; both breast size and shape often did become abnormal when EE2 was used. However, this occurred only rarely when the more recent transdermal estradiol low-dose regime was used (personal communications and observations).

Treatment regimens for both GH and estrogen have changed since these trials were planned and initiated. The dose of GH given routinely to girls with TS is now closer to the range observed in the present study. Moreover, ERT is now primarily given in the form of 17β-estradiol, and the administration route is usually transdermal. Furthermore, the dose of estrogen used to induce puberty has reduced substantially, dose and tempo of administration should ideally mimic the initially very low serum estradiol levels of normal female puberty (38, 39). For maintenance, a dose should be used sufficient to result in serum levels appropriate for the young adult women, a dose that is twice that recommended for post-menopausal women (40–42).

Of all 144 girls with TS enrolled in this study, 37 girls experienced onset of puberty spontaneously; 5 of those received LHRH analogue treatment for a period (note: the data of these five girls were excluded before any analyses), and 10 other girls needed after some time ERT in order to undergo full puberty with menarche and sustained development of secondary sexual characteristics. Thus, 22 girls went through a full spontaneous puberty with regular menses. They started puberty at age mean 11.8 years, significantly younger than those who started puberty spontaneously but later needed ERT. Pubertal height gain was also greater in the former group, 12.0–17.5 cm, when receiving the high GH dose. Most of the girls who underwent full spontaneous puberty had a karyotype labelled as “other” (i.e., structural aberrations); this karyotype was also found to be positively associated with pubertal height gain, possibly indicating more subtle features of TS. In contrast, girls with TS with later spontaneous puberty grow less during infancy and mid-childhood, probably estrogen dependent (76). The height gain observed in those undergoing spontaneous puberty in these studies could serve as a future treatment goal for girls with TS needing ERT. Generating serum estradiol concentrations that mirror those seen physiologically during puberty in girls without TS would theoretically be optimal (38, 59). This approach has been tested in girls with TS receiving ERT in combination with GH, and it resulted in a growth-promoting effect (26, 27).

### 5.4 Oxandrolone as adjuvant growth-promoting treatment

Almost all girls, 94%, in GH_old_ groups received adjuvant oxandrolone treatment compared with only half of the GH_young_ group. The growth stimulating effect of oxandrolone is well documented (10). In recent reviews and a meta-analysis, the additive effect on growth was calculated to be 2.3–4.6 cm (77) and 2.06 cm (78), respectively. The dose used in the present studies was consistent with those described in these more recent publications. However, in the multivariable analysis, oxandrolone treatment was not found to be a predictor of growth in SDS, possibly due to the high proportion of study subjects receiving treatment. When calculating growth in centimeters, there was a small negative impact on growth, indicating interaction with the maturation tempo or, more likely, a selection bias owing to the use of oxandrolone. A possible long-term negative effect of oxandrolone treatment in adolescence on QoL and socio-emotional functioning in adulthood has been identified (79), which may oblige us to optimize GH treatment to minimize oxandrolone treatment, thereby also avoiding negative effects on mammary development and the risk of voice deepening and other virilising effects (77). Thus, early diagnosis of TS and initiation of GH treatment at a suitable dose could allow us to normalize height in time to allow ERT to be initiated in harmony with the onset of puberty in peers (7, 37).

### 5.5 Methodological aspects on evaluation of height during childhood and puberty

The goal for height-promoting therapy is normalization. The girls themselves compare heights both with their family and their non-TS peers. Therefore, we primarily used the non-TS reference when calculating SDS (6), which includes the childhood component calculated from the ICP-growth model (3). This prepubertal growth function within this model allows height gain calculations in SDS to be made separately for growth related to the prepubertal phase, from the specific pubertal growth. Thereby, the influence of any normal early pubertal growth in the reference population was omitted.

As age at start of puberty/ERT and age at AH have great variations within the groups and this interferes with SDS calculations, it is necessary to use centimeter when discussing total pubertal height gain. The effect of different timing of pubertal growth in the reference population and in our treated TS girls is visualized in **Figure 5**. Until recently, only data on the entire pubertal period could be used for calculations of pubertal change in height_SDS_ (6, 80). However, a novel type of pubertal growth reference, aligned for height at the onset of puberty, is now available (81). This height reference will allow comparisons in SDS and centimeter at any timepoint throughout the pubertal period and serve as a tool for monitoring the impact of treatment with GH on growth during puberty, for both total pubertal growth and separating ongoing basic growth from the specific pubertal growth, including also references for weight and BMI (82, 83). However, for this report, calculation of AH_SDS_ was adjusted by aligning AH for girls with TS in centimeters with height at 18 years for the reference population (6). When we also compared heights with our TS height reference (4, 5), the entire treatment effect, the total height gain, was approximately 2 SDS for girls with TS on the low GH dose and approximately 3 SDS for those on the high GH dose. Thus, reducing on the low dose approximately half of the deficit relative to their parental heights and almost all deficit on the high GH dose (**Table 4**, **Figures 2**, **3**).

### 5.6 Strengths and limitations

The major strength of these studies was that they are based on national-level data collected over a considerable time period. All Swedish pediatric endocrinologists and pediatricians caring for patients with TS participated, resulting in over 25 years of clinical follow-up from a range of healthcare professionals within the multidisciplinary team. The setting also allowed researchers to gather knowledge on the management of TS and led to the development of tools for monitoring treatment efficacy and safety in clinical practice.

#### 5.6.1 Study population

Out of all those diagnosed in Sweden with TS 1987–1998, a “homogeneous” study population was obtained: with known karyotype for the participants and with inclusion/exclusion criteria narrowing the study group by avoiding those with Y-line and those with severe organ diseases.

#### 5.6.2 The study design

The study design, being an open temporal design, allows generalization of the results, as it includes all girls diagnosed with TS in the country fulfilling the criteria. All university and regional pediatric clinics in Sweden and most local ones were involved, thus making the study as close as possible to the real-world situation. While this was beneficial in many ways, and the results obtained in this situation highlight the clinical need for GH dose to be individualized, it was not possible to control dosing levels or to control for unknown events over time, possible with other types of design. However, it is important to remember that there were other ongoing studies looking at individualization of GH dose at the time of these investigations (84, 85). Swedish pediatric endocrinologists were therefore well acquainted with working clinically according to this dose-changing concept. This experience may have influenced the dose adaptations made for the TS girls.

#### 5.6.3 National knowledge gathering led to development of tools for monitoring safety and efficacy

##### 5.6.3.1 Centralized laboratory analyses

Centralized laboratory analyses were used for all hormonal pre-investigation and follow-up of efficacy and safety variables. Extensive laboratory monitoring throughout the studies ensured that care could be changed as needed and made it possible to gather unique new knowledge about girls with TS: different GH profiles (51), different GH forms (65, 66), GHBP (52), different FSH forms (54, 86, 87), and more to come through the biobank, and for autoimmune diseases as celiac disease (55) and thyroid hormone disturbances (53).

##### 5.6.3.2 Proper growth evaluation

To make it possible, we developed both a height reference (5) and an ICP growth model (4) by using data on spontaneous growth in girls diagnosed with TS. Both items were developed using methods comparable to those used to create the reference and growth model developed from healthy girls (6, 88).

#### 5.6.4 Variables followed within the study

All yearly follow-up visits were at the university hospitals, with yearly X-ray bone age (10). Most girls were visiting the GP-GRC as the national center for TS, and QoL and psychological functioning of the girl with TS (89, 90) and the psychosocial impact on her family (91, 92) were explored.

##### 5.6.4.1 Voice frequency

Voice frequency was recorded, and follow-up showed speech frequency to be normalized on GH and oxandrolone treatment (93, 94).

##### 5.6.4.2 Hearing function and ear

Also followed were ear and hearing problems, ranging from external morphological abnormalities to sensorineural or conductive hearing loss, known to constitute major medical issues affecting the QoL and wellbeing in girls and women with TS (95). The prevalence of otological disease as external ear deformities (20–62%), recurrent otitis media (24–48%), and hearing loss (36–84%) is high in TS (96). When subdivided according to karyotype, 45,X and “45,X/46,iso(X) and equivalents” (i.e., TS harboring an isochromosome) experience hearing loss, middle ear infections, and external ear malformations more often than other karyotypes (97).

Hearing correlate to height and IGF-1 concentrations (98). Thus, it was hypothesized that lacking the p-arm is detrimental for the TS phenotype, which could be due to growth defects, attributed to a combination of the generally prolonged cell cycle time in abnormal chromosomal cells and the haploinsufficiency of growth-regulating Xp-linked genes, such as SHOX (98). So far, no impact of ERT nor of GH on these conditions has been shown (99, 100), as supported by the results of our study. The hearing loss with age did not differ between the two GH dose groups (101).

#### 5.6.5 Variables not followed within the study

Despite the collection of these many variables, bone quality and uterine growth were not followed as part of the trials. Initiated by those who were enrolled in these trials, there was structured follow-up by the Turner Academia teams at the university hospitals for young women with TS after transition to adult care. Thus, we will be able to investigate the impact of the different treatment regimens on these variables.

##### 5.6.5.1 GH and ERT for bone mineralization and bone health

A more physiological endocrine milieu would favor both normal growth and optimal bone mineralization, helping girls to attain a normal peak bone mass (PBM). Today, we are aware of the favorable effect of GH on amplitude and timing of PBM (102) and the role this has in reducing the risk of later osteoporosis. Unfortunately, GH therapy was ended prematurely in the girls participating in our studies; one-third ended GH treatment too early for growth, and all girls ended according to the protocol when growth velocity was still 2 cm/year, which was before the attainment of PBM. In addition, it is known that starting ERT late delays bone mass accrual (103, 104) and note that the “pediatric” adult ERT dose at that time of these studies was only 25 µg/kg/day of transdermal estradiol, i.e., a suboptimal dose for a young adult woman. However, the route of administration was beneficial: compared with oral EE2, transdermal estradiol was found to result in faster bone accrual in the spine (105).

##### 5.6.5.2 GH and ERT for growth of uterus

There are doubts about estrogen dosing in these trials. Was it too high in the beginning hampering pubertal growth and was it too low after attained AH with insufficient effects on uterus and bone? Could uterine growth and size have guided us to identify optimal individual dose of ERT? Our rational for this question is that both GH and estrogen are uterine and endometrium growth promoters (106–108). Prepubertal GH treatment increases uterine size (109) and may positively prepare the uterus for the effects of pubertal ERT. Estradiol dose has implications for uterine size: in a post-menarcheal group uterine length reflected estrogen dose (110). In addition, in girls with TS for whom puberty was induced, uterine size was small compared with those who underwent spontaneous puberty (111), as was uterine size in estradiol-treated TS small women compared with non-TS healthy women (112), while a more normal size was found by others (113). Thus, uterine size reflects the estrogen effect on uterus and possibly could monitor and guide individual dosing. However, there is a conflict between the low estradiol dose promoting pubertal growth and the higher dose stimulating uterine growth. Thus, we should aim for a low enough growth-stimulating estradiol dose in early puberty, to be followed during puberty by an estradiol dose high enough to promote normal uterine size. If uterine size post-menarche is found too small, an increased estradiol dose can stimulate uterine growth to obtain normal adult size (44, 110, 114), as increasing fertility possibilities makes uterine size and shape important (115–117).

#### 5.6.6 Safety aspects

We aimed to mirror normal physiology using the hormones given and thereby minimize the occurrence of under- and overtreatment effects. There were no SAEs of diabetes, thrombosis, or increased intracranial pressure reported in association with treatment. Five girls withdrew from the study after enrollment owing to other diseases that were reported as AEs.

*GH*. Girls who were planned to receive GH dose 67 µg/kg/day started treatment with the lower dose, with dose escalations being made over 1–3 months; in a few cases, dose escalation was slower than planned, mostly due to a tendency towards edema. In practice, both GH doses were not always maintained; the GH_67_ dose was often reduced, while the GH_33_ dose was increased. Probably when the investigators observed signs of overdose or lack of efficacy on growth, then the dose was individually adapted.

*Oxandrolone*. Oxandrolone was used from 11 years age in almost all girls in the GH_old_ group and in about half of the GH_young_ group. Voice deepening is a well-known effect of oxandrolone (77) and if observed/reported, led to prompt dose reduction as was also done when virilization and delayed breast development were seen (118); however, these effects were rarely seen in our study.

*Oral estrogen*. Oral estrogen, EE2, was used for many years in the present studies. Even though this synthetic, long-acting form is known to impact on coagulation factors and metabolism due to its liver passage and that levels in serum cannot be reliably estimated to protect against overdosing, no clinically significant AEs were seen. Similarly, no AEs were reported in connection with the use of 17β-estradiol in the present study; 17β-estradiol was administered via the transdermal route, which is known to circumvent liver passage and thereby avoids cardiometabolic side effects (105, 119).

### 5.7 Transition

#### 5.7.1 Pediatric studies highlighted the need for organized care in a lifelong perspective

Before clinical trials on GH treatment, healthcare for girls and women with TS was managed by various different clinics and primary care practitioners, resulting in late diagnoses and high study/treatment dropout rates.

The GH trials led to a worldwide shift towards the centralization of care for children with growth disorders, including those with TS to pediatric endocrinologists working within university hospitals and national centers such as GP-GRC in Sweden, responsible for both clinical care and research. Here, multidisciplinary teams for the girls with TS were formed, including both organ, function, and growth phase specialties, during the transition years closely together with the fertility gynecologist.

During these long-term studies, a need was identified for a lifelong, patient-centered approach to the care of these individuals, which saw the extension of care from childhood through adolescence to adulthood. The fertility gynecologist, who had gained knowledge by participating during transition, as the key person working closely with the endocrinologist formed the multidisciplinary team with specialists in essential disciplines after transition to maintain a lifelong perspective (120). The practical approach with “TS days” is that it should be both convenient for the TS women to meet the specialists they need and for the specialists in different areas to meet for sharing knowledge, which is proven to be a successful concept (51, 52). This has been advocated within the Swedish Turner Academy organization, initiated after the 4th TS meeting in Gothenburg in 1995, devoted to TS in a lifespan perspective (121). The concept of international TS consensus meeting was initiated (121), with subsequent meetings being held in 2001 (122), 2007 (104), and most recently 2017 (37). As a result, care for girls and women with TS continues to improve, and international guidelines and recommendations for the management of TS are continuously updated.

#### 5.7.2 Education and independence training during the transition phase

Today, the pediatric endocrinology team often plays a key role in coordinating care provided by other specialists during childhood. With improvements in medical knowledge, the number of areas and therapies that this multidisciplinary team needs to monitor has increased considerably. Not least important are the social and educational aspects, including QoL and self-esteem obtained during childhood and adolescence, as they will have lifelong implications.

The transition between pediatric and adult care is a critical process that takes place over several years. Both the pediatric endocrinologist and adolescent/fertility gynecologist are involved, and alongside this, each young girl will take on increasing responsibilities for herself. This “independence” training is important if we are to prevent the well-documented high treatment dropout rates in young adulthood and needs to start years before the girl becomes a legal adult. In dialogue with her future health providers, discussions with each girl about transition should cover not only all aspects of her present health and social situation but also possible problems that may arise with age, thereby minimizing the risk of low therapy adherence in adulthood.

Patients typically request a known contact path, with easy access to the team and a key specialist within the multidisciplinary team who is well trained in TS healthcare. This person must be able to guide the individual and facilitate connectivity within the healthcare system if or when a new sign or symptom presents. Because TS health issues are complex, the involvement of various kinds of specialists and their multidisciplinary expert team is crucial. Easy interdisciplinary communication is of foremost importance not only for care but also for the continued expansion of knowledge about this syndrome, which will bring further benefits to the TS girl or woman.

Thus, bearing in mind the complexity of TS, with the potential for major health issues impacting most organ systems, the management should not sit within primary care.

### 5.8 Future challenges

We identified three essential challenges.

First is early diagnosis. Still, early diagnosis of TS remains a challenge (123). Early identification is essential if we are to normalize growth during childhood, with the benefits that this will bring in terms of QoL and wellbeing. Thus, the observation of a significant difference in height_SDS_ relative to mid-parental height (diffSDS) should raise the suspicion of TS and prompt referral for karyotyping

Although we have shown using current treatment regimens that most girls with TS can achieve a normal prepubertal height, and an AH within the normal range, it is obvious that growth during puberty remains suboptimal. As a result, the induction of puberty in girls with TS is often delayed until a point at which the individual is happy with her current height; this may be quite a bit later than the spontaneous onset of puberty in her peers. If we can diagnose and start GH treatment at an earlier age, it will provide the opportunity to normalize height while the individual is younger, thus allowing normal height during childhood and puberty to be induced at a more normal age.

Second is normalize puberty. Finding a way to improve pubertal growth for girls with TS remains also as a challenge. How should this be improved? The GH dose may need to more closely mimic the three–fourfold increment seen in puberty in non-TS girls (64). ERT may be optimized for better growth stimulation regarding gain and tempo/duration while still ensuring normal development of secondary sex characteristics (58). There are also other unanswered questions, such as possible substitution of the androgen deficiency (124). Whatever the direction of future studies on pubertal growth, evaluations will be facilitated from the newly developed growth models on pubertal growth references that enable individual monitoring of growth, such as height (81) weight, and BMI (82, 83), in an individual during the pubertal period relative to growth of individuals of a healthy population, aligned for the onset of puberty, spontaneously or at start of ERT.

Third is the lifelong structured follow-up. The challenge will be to study the long-term effects during lifespan of the combined administration of GH and ERT in women with TS. In particular, it is of interest to study possible hormone-sensitive age-related conditions such as hearing and balance, fracture risk, metabolic or cardiovascular diseases, and dementia. Follow-up investigations are already being conducted in women with TS, and hopefully, these will provide insights that will help us to shape future care.

### 5.9 Conclusion

This study of different GH doses on growth and puberty in girls with TS shows a clear dose effect on not only increased heights but also earlier ages for pubertal onset and AH. It highlights the importance of using a high GH dose when starting treatment to maximize prepubertal height gain and normalize childhood growth for optimal QoL and self-esteem from childhood onwards. Although the successful prepubertal gain was partly lost during puberty, an AH within the normal range was achieved, however, at the cost of both delayed pubertal development and attained AH. Of note, pubertal height gain was substantially lower in estradiol-treated girls with TS compared with those who underwent a spontaneous puberty. These findings highlight a need to improve pubertal GH and ERT regimens and to monitor changes during puberty more closely to this end, to achieve a more normal pubertal growth spurt, peak bone mass, and uterine size before transition. Future studies including individual GH dosing and growth-promoting pubertal induction and maintaining strategies in girls with TS are warranted, during the critical window possible in time, knowing the importance for a pubertal development as normal as possible for psychosocial health, hearing, possible future fertility intervention, bone, and cardiovascular health.

## Data availability statement

The raw data supporting the conclusions of this article will be made available by the corresponding author, without undue reservation.

## Ethics statement

The studies involving human participants were reviewed and approved by Ethical Committees of Sweden at the university hospitals in Lund (221/87), Gothenburg, Linköping, Umeå, Uppsala (all 76-88) and the Karolinska Institute (88-40). Written informed consent from the participants’ legal guardian/next of kin was not required to participate in this study in accordance with the national legislation and the institutional requirements.

## Author contributions

KON was the initial principal investigator; in 2014, he handed over to KA-W as principal investigator for these national TS investigator-initiated clinical trials (TRN 87-052-01 and TRN-072). Co-investigators from 1987 onwards were KA-W and from 1991 BK. KON and KA-W contributed to the study design. CA-L and KA-W wrote the statistical analysis plan and the first draft of the manuscript. BK, CA-L, M-LB, and KA-W contributed to the interpretation and analysis of data. BK, CA-L, M-LB, and KA-W contributed to the writing and revising of the manuscript for important intellectual content. All authors contributed to the article and approved the submitted version.
