# Supplementary material for: Normalization of puberty and adult height in girls with Turner syndrome: results of the Swedish Growth Hormone trials initiating transition into adulthood
Source: Front Endocrinol (Lausanne). 2023 Jul 17;14:1197897. doi: 10.3389/fendo.2023.1197897 (PMC10389045; doi:10.3389/fendo.2023.1197897)
Supplement: Supplementary file 1 [file DataSheet_1.pdf]

Supplementary material to

Normalization of puberty and adult height in girls with Turner syndrome: results of the Swedish Growth Hormone trials initiating transition into adulthood

Berit Kriström 1†, Carina Ankarberg-Lindgren 2†, Marie-Louise Barrenäs 3, Karlolof Nilsson 4, and Kerstin Albertsson-Wikland 3*

[1]Department of Clinical Science, Pediatrics, Umeå University, Umeå, Sweden

[2]Institute of Clinical Sciences, Department of Pediatrics, Göteborg Pediatric Growth Research Center, Sahlgrenska Academy, University of Gothenburg, Gothenburg, Sweden

[3]Institute of Neuroscience and Physiology, Department of Physiology/Endocrinology, Sahlgrenska Academy, University of Gothenburg, Gothenburg, Sweden

[4]Department of Clinical Sciences, University Hospital Malmö, Lund University, Malmö, Sweden

[†]**Equal contribution and first authorship:** These authors contributed equally to this work and share first authorship.

***Correspondence:**

Kerstin Albertsson-Wikland, kerstin.albertsson.wikland@gu.se

Supplementary Tables page

References 2

Comparison between GH doses per age group PP: 3-8
Table 1a Pretreatment characteristics, **PP** population, **young** vs dose 3
Table 1b Auxology from GHstart to PO, PP population, young vs dose 4
Table 1c Auxology at adult height, PP population, young vs dose 5

Table 2a Pretreatment characteristics, **PP** population, **old** vs dose 6
Table 2b Auxology from GHstart to PO, PP population, old vs dose 7
Table 2c Auxology at adult height, PP population, old vs dose 8

Comparison between GH doses for both age groups ITT: 9-11
Table 3a Pretreatment characteristics, ITT population, **all girls vs dose** 9
Table 3b Auxology from GHstart to PO, ITT population, all girls vs dose 10
Table 3c Auxology at adult height, ITT population, all girls vs dose 11

Comparison between ages per GH dose ITT: 12-14
Table 4a Pre-treatment characteristics, ITT population, **all girls vs age** 12
Table 4b Auxology from GH-start to PO, ITT population, all girls vs age 13
Table 4c Auxology at adult height, ITT population, all girls vs age 14

Table 5 Auxology from birth to adult height, PP population, **Turner Reference** 15

Comparison between GH doses for both age groups PP: 16-18
Table 6a Pretreatment characteristics, PP population, **all girls vs dose** 16
Table 6b Auxology from GH-start, PP population, all girls vs dose 17
Table 6c Auxology at adult height, PP population, all girls vs dose 18

Comparison between ages per GH dose PP: 19-21
Table 7a Pretreatment characteristics, PP population, **all girls vs age** 19
Table 7b Auxology from GH-start, PP population, all girls vs age 20
Table 7c Auxology at adult height, PP population, all girls vs age 21

References:

1. **Niklasson A & Albertsson-Wikland, K:** Continuous growth reference from 24th week of gestation to 24 months by gender. *BMC Pediatrics 2008;8:8*
2. **Albertsson-Wikland, K**, Luo ZC, Niklasson A, Karlberg J: Swedish population-based longitudinal reference values from birth to 18 years of age for height, weight, and head circumference. *Acta Paediatrica 2002;91: 739-754.*
3. **Luo ZC,** Albertsson-Wikland K, Karlberg J: Target height as predicted by parental heights in a population-based study. Pediatric Research 1998;44:563-571.
4. **Karlberg J**: on the construction of the infancy-childhood-puberty growth standard. *Acta Paediatrica Scandinavia1989;356:26-37*.
5. **Karlberg J,** Albertsson-Wikland K, Weiss Naeraa R: The Infancy-Childhood-Puberty (ICP) Model of Growth for Turner Girls. *Excerpta Medica ICS 1991;924:89-94.*
6. **Rongen-Westlaken C,** Corel L, van den Broeck J, Massa G, Karlberg J, Albertsson-Wikland K, Naeraa RW, Wit JM: Reference values for height, height velocity, and weight in Turner 's syndrome. *Acta Paediatrica 1997;86:937-942.*

Suppl Table 1a. Pre-treatment characteristics for the **young** subgroup versus dose in the **PP** population versus Swedish references {1,2,3,4}.

Variables	Dose 33 µg (n=25)	Dose 67 µg (n=28)	p-value	Difference between groups Mean (95% CI)	Effect Size
**Karyotype 45X**	20 (80.0%)	15 (53.6%)			
**Mosaic**	1 (4.0%)	1 (3.6%)			
**Other**	4 (16.0%)	12 (42.9%)	0.085		
**Missing**					
**At birth**					
**GA (weeks)**	38.7 (1.6)<br>39 (36; 42)<br>n=24	38.7 (2.0)<br>39 (35; 42)<br>n=27	1.00	-0.037<br>(-1.077; 1.000)	0.021
**Length (SDS)**	-2.48 (1.53)<br>-2.46 (-5; 0.9)<br>n=25	-2.07 (1.16)<br>-2 (-4.34; 0.23)<br>n=28	0.27	-0.416<br>(-1.151; 0.330)	0.309
**Weight (SDS)**	-1.50 (1.47)<br>-1.27 (-5.11; 0.37)<br>n=25	-1.28 (1.20)<br>-0.87 (-3.92; 0.18)<br>n=28	0.56	-0.216<br>(-0.946; 0.532)	0.162
**Mother height (SDS)**	-0.239 (0.876)<br>-0.341 (-1.504; 0.997)<br>n=25	-0.017 (1.265)<br>-0.142 (-2.078; 2.333)<br>n=28	0.47	-0.222<br>(-0.835; 0.376)	0.202
**Father height (SDS)**	-0.228 (0.875)<br>-0.255 (-1.79; 1.434)<br>n=25	-0.303 (1.233)<br>-0.332 (-3.326; 1.398)<br>n=28	0.80	0.075<br>(-0.502; 0.682)	0.070
**MPH (SDS)**	-0.290 (0.828)<br>-0.458 (-2.046; 1.185)<br>n=25	-0.199 (1.253)<br>-0.363 (-3.356; 2.066)<br>n=28	0.76	-0.091<br>(-0.683; 0.499)	0.085
**DiffMPH (SDS)**	-2.19 (1.76)<br>-2.17 (-5.79; 1.48)<br>n=25	-1.87 (1.29)<br>-1.91 (-4; 0.9)<br>n=28	0.44	-0.325<br>(-1.163; 0.527)	0.212
**At GH-start**					
**Pre treatment height velocity, the years before (cm/year)**	5.46 (1.24)<br>5.49 (3.41; 8.12)<br>n=25	5.36 (1.30)<br>5.47 (2.51; 7.41)<br>n=27	0.79	0.091<br>(-0.620; 0.810)	0.072

For categorical variables n (%) is presented.
For continuous variables Mean (SD) / Median (Min; Max) / n= is presented.
For comparison between groups Chi Square Exact test was used for non-ordered categorical variables and the Fisher´s Non Parametric Permutation Test was used for continuous variables.
The confidence interval for the mean difference between groups is based on Fishers non-parametric permutation test.
Effect size is absolute difference in mean / pooled SD.
GA, gestational age; GH, growth hormone; SDS, standard deviation score; MPH, mid-parental height; DiffMPH, difference in SD score between the height of the girl and the heights of her parents.

Suppl Table 1b. Auxology from GH-start for the **young** subgroup versus dose for the **PP** population versus Swedish references {1,2,3,4}.

variabler	Dose 33 µg (n=25)	Dose 67 µg (n=28)	p-value	Difference between groups Mean (95% CI)	Effect Size
**At GH start**					
**Age (yrs)**	5.83 (1.41)<br>5.52 (3.04; 8.61)<br>n=25	5.81 (1.79)<br>5.74 (3.15; 8.89)<br>n=28	0.98	0.015 (-0.894; 0.921)	0.009
**Height$_{SDS}$**	-2.80 (0.73)<br>-2.84 (-3.97; -1.13)<br>n=25	-2.86 (0.81)<br>-2.81 (-5.39; -1.52)<br>n=28	0.77	0.059 (-0.362; 0.496)	0.076
**Diff MPH$_{SDS}$**	-2.51 (0.91)<br>-2.35 (-4.12; -0.56)<br>n=25	-2.66 (0.97)<br>-2.52 (-4.5; -1.08)<br>n=28	0.56	0.150 (-0.364; 0.668)	0.160
**+ 1 yr All prepubertal**					
**Average GH dose (µg/kg/day)**	35.1 (3.9)<br>35.5 (25.5; 41.6)<br>n=25	54.3 (6.0)<br>54.9 (39.4; 66.4)<br>n=28	<.0001	-19.2 (-22.1; -16.4)	3.75
**Height$_{SDS}$**	-2.27 (0.74)<br>-2.26 (-3.57; -0.66)<br>n=25	-2.00 (0.79)<br>-1.9 (-4.35; -0.51)<br>n=28	0.22	-0.269 (-0.691; 0.161)	0.349
**At puberty start**					
**Age (yrs)**	14.6 (1.2)<br>14.5 (12.5; 16.9)<br>n=25	12.8 (1.2)<br>13.1 (10.6; 14.9)<br>n=28	<.0001	1.80 (1.13; 2.44)	1.52
**Height$_{SDS}$**	-0.990 (0.769)<br>-0.962 (-2.645; 0.267)<br>n=25	-0.513 (1.026)<br>-0.407 (-3.022; 1.258)<br>n=28	0.067	-0.477 (-0.980; 0.033)	0.521
**Diff MPH$_{SDS}$**	-0.700 (0.909)<br>-0.528 (-2.791; 0.798)<br>n=25	-0.314 (1.045)<br>-0.263 (-2.412; 1.794)<br>n=28	0.16	-0.386 (-0.923; 0.159)	0.392
**Average GH dose Pre puberty (µg/kg/day)**	34.0 (1.7)<br>33.8 (30.8; 38.4)<br>n=25	58.8 (4.3)<br>59.5 (46.7; 65.4)<br>n=28	<.0001	-24.8 (-26.6; -22.9)	7.38
**Gain in height$_{SDS}$ GH start - Puberty onset**	1.81 (0.62)<br>1.71 (0.61; 3.4)<br>n=25	2.34 (0.79)<br>2.29 (0.81; 4.38)<br>n=28	0.0086	-0.536 (-0.926; -0.140)	0.747

For continuous variables Mean (SD) / Median (Min; Max) / n= is presented.
For comparison between groups the Fisher´s Non Parametric Permutation Test was used for continuous variables.
The confidence interval for the mean difference between groups is based on Fishers non-parametric permutation test.
Effect size is absolute difference in mean / pooled SD.
GH, growth hormone; SDS, standard deviation score; MPH, mid-parental height; DiffMPH, difference in SD score between the height of the girl and the heights of her parents.

Suppl Table 1c Auxology at adult height for the **young** subgroup versus dose in the **PP** population versus the Swedish references [1,2,3,4]..

Variables	Dose 33 µg (n=25)	Dose 67 µg (n=28)	p-value	Difference between groups Mean (95% CI)	Effect Size
**At adult height**					
**Age (yrs)**	17.3 (1.2)<br>17 (15.6; 21.4)<br>n=25	16.3 (1.1)<br>16.1 (14.7; 19.8)<br>n=28	0.0015	1.06 (0.41; 1.72)	0.891
**Adult height (cm)**	154.3 (4.9)<br>155.5 (146; 165.4)<br>n=25	157.0 (6.6)<br>157.2 (145.1; 168.3)<br>n=28	0.11	-2.67 (-5.95; 0.62)	0.456
**$Height_{SDS}$**	-2.18 (0.81)<br>-1.99 (-3.55; -0.36)<br>n=25	-1.74 (1.08)<br>-1.71 (-3.7; 0.12)<br>n=28	0.11	-0.440 (-0.978; 0.102)	0.456
**Diff MPH_{SDS}**	-1.89 (0.81)<br>-1.84 (-3.58; -0.67)<br>n=25	-1.54 (1.02)<br>-1.51 (-4.07; 0.49)<br>n=28	0.18	-0.349 (-0.858; 0.167)	0.377
**Gain in $Height_{SDS}$ Puberty onset - Adult height**	-1.19 (0.39)<br>-1.17 (-1.88; -0.57)<br>n=25	-1.22 (0.47)<br>-1.26 (-2.08; -0.54)<br>n=28	0.75	0.037 (-0.200; 0.274)	0.086
**Gain in height (cm) Puberty onset - Adult height**	3.51 (2.82)<br>2.8 (-0.2; 10.1)<br>n=25	9.09 (5.44)<br>7.9 (0.8; 20.5)<br>n=28	<.0001	-5.58 (-7.99; -3.13)	1.27
**Average GH $dose_{Total}$ (µg/kg/day)**	34.0 (1.8)<br>33.7 (31; 38.3)<br>n=25	57.9 (4.4)<br>58 (46.6; 65.9)<br>n=28	<.0001	-23.9 (-25.8; -22.0)	7.02
**Time on GH (yrs)**	10.4 (1.6)<br>10.3 (7.2; 12.6)<br>n=25	9.43 (2.02)<br>9.72 (5.36; 13.87)<br>n=28	0.068	0.944 (-0.072; 1.958)	0.518
**Duration puberty (yrs)**	2.72 (1.08)<br>2.68 (0.98; 5.28)<br>n=25	3.45 (1.64)<br>3.31 (1.02; 9.26)<br>n=28	0.064	-0.735 (-1.507; 0.039)	0.523
**Gain in $height_{SDS}$ GH start - Adult height**	0.620 (0.679)<br>0.646 (-0.694; 1.691)<br>n=25	1.12 (0.79)<br>1 (-0.52; 3.27)<br>n=28	0.016	-0.499 (-0.908; -0.092)	0.672

For continuous variables Mean (SD) / Median (Min; Max) / n= is presented.
For comparison between groups the Fisher´s Non Parametric Permutation Test was used for continuous variables.
The confidence interval for the mean difference between groups is based on Fishers non-parametric permutation test.
Effect size is absolute difference in mean / pooled SD.
GH, growth hormone; SDS, standard deviation score; MPH, midparental height; DiffMPH, difference in SD score between the height of the girl and the heights of her parents.

Suppl Table 2a. Pre-treatment characteristics for the **old** subgroup versus dose in the **PP** population versus Swedish references {1,2,3,4}.

Variables	Dose 33 µg (n=23)	Dose 67 µg (n=13)	p-value	Difference between groups Mean (95% CI)	Effect Size
**Karyotype 45X**					
**Mosaic**	7 (30.4%)	8 (61.5%)			
**Other**	1 (4.3%)	0 (0.0%)			
**Missing**	15 (65.2%)	5 (38.5%)	0.20		
**At birth**					
**GA (weeks)**	39.3 (1.5)<br>39 (36; 42)<br>n=22	38.6 (1.6)<br>39 (36; 41)<br>n=11	0.31	0.636 (-0.500; 1.778)	0.427
**Length (SDS)**	-2.11 (1.59)<br>-1.93 (-5.49; 0.97)<br>n=23	-2.13 (1.16)<br>-1.86 (-4.48; -0.35)<br>n=12	0.96	0.028 (-1.001; 1.062)	0.019
**Weight (SDS)**	-1.69 (1.21)<br>-1.39 (-4.73; 0.42)<br>n=23	-1.27 (1.12)<br>-0.98 (-3.07; 1.41)<br>n=13	0.32	-0.418 (-1.250; 0.416)	0.355
**Mother height (SDS)**	-0.294 (0.665)<br>-0.182 (-1.456; 1.093)<br>n=23	-0.064 (0.876)<br>-0.182 (-1.775; 1.571)<br>n=13	0.38	-0.230 (-0.756; 0.305)	0.308
**Father height (SDS)**	-0.083 (0.909)<br>-0.101 (-1.713; 1.818)<br>n=22	0.186 (0.723)<br>0.144 (-1.023; 1.588)<br>n=13	0.38	-0.269 (-0.858; 0.328)	0.318
**MPH (SDS)**	-0.273 (0.705)<br>-0.105 (-1.504; 0.873)<br>n=22	0.076 (0.739)<br>0.001 (-1.547; 1.058)<br>n=13	0.18	-0.349 (-0.855; 0.157)	0.486
**DiffMPH (SDS)**	-1.84 (1.64)<br>-1.57 (-5.89; 0.96)<br>n=22	-2.34 (1.22)<br>-2.39 (-4.4; -1.03)<br>n=12	0.36	0.504 (-0.597; 1.590)	0.334
**At GH-start**					
**Pre treatment height velocity, the years before (cm/year)**	3.62 (0.97)<br>3.55 (1.48; 5.01)<br>n=23	3.76 (0.65)<br>3.84 (2.82; 4.5)<br>n=10	0.70	-0.140 (-0.826; 0.517)	0.158

For categorical variables n (%) is presented.
For continuous variables Mean (SD) / Median (Min; Max) / n= is presented.
For comparison between groups Chi Square Exact test was used for non-ordered categorical variables and the Fisher´s Non Parametric Permutation Test was used for continuous variables.
The confidence interval for the mean difference between groups is based on Fishers non-parametric permutation test.
Effect size is absolute difference in mean / pooled SD.
GA, gestational age; GH, growth hormone; SDS, standard deviation score; MPH, mid-parental height; DiffMPH, difference in SD score between the height of the girl and the heights of her parents.

Suppl Table 2b. Auxology from GH-start for the **old** subgroup versus dose for the **PP** population versus Swedish references {1,2,3,4}.

variabler	Dose 33 µg (n=23)	Dose 67 µg (n=13)	p-value	Difference between groups Mean (95% CI)	Effect Size
**At GH start**					
**Age (yrs)**	11.8 (2.0)<br>11.2 (9; 15.9)<br>n=23	11.4 (1.6)<br>10.7 (9.2; 14.8)<br>n=13	0.61	0.331 (-0.931; 1.645)	0.179
**$Height_{SDS}$**	-2.83 (0.76)<br>-2.74 (-4.43; -1.36)<br>n=23	-2.82 (0.57)<br>-2.93 (-3.96; -1.44)<br>n=13	0.98	-0.007 (-0.490; 0.479)	0.011
**Diff MPH_{SDS}**	-2.55 (0.80)<br>-2.44 (-3.86; -0.86)<br>n=22	-2.89 (0.81)<br>-2.86 (-4.01; -1.44)<br>n=13	0.22	0.348 (-0.231; 0.920)	0.434
**+ 1 yr All prepubertal**					
**Average GH dose (µg/kg/day)**	33.4 (3.3)<br>33.5 (26.7; 40)<br>n=23	56.5 (4.1)<br>56.1 (48.2; 62.5)<br>n=13	<.0001	-23.2 (-25.7; -20.6)	6.41
**$Height_{SDS}$**	-2.27 (0.79)<br>-2.35 (-3.87; -0.81)<br>n=20	-1.75 (0.61)<br>-1.77 (-2.83; -0.41)<br>n=13	0.058	-0.518 (-1.048; 0.018)	0.714
**At puberty start**					
**Age (yrs)**	15.0 (1.6)<br>15.3 (11.2; 16.8)<br>n=23	14.1 (1.7)<br>14.4 (11.4; 16.6)<br>n=13	0.12	0.910 (-0.248; 2.025)	0.565
**$Height_{SDS}$**	-1.40 (0.52)<br>-1.52 (-2.28; -0.43)<br>n=23	-0.776 (0.607)<br>-0.575 (-1.771; -0.065)<br>n=13	0.0022	-0.624 (-1.023; -0.225)	1.13
**Diff MPH_{SDS}**	-1.14 (0.79)<br>-1.44 (-2.14; 0.67)<br>n=22	-0.851 (0.679)<br>-0.943 (-1.845; 0.353)<br>n=13	0.29	-0.285 (-0.815; 0.266)	0.378
**Average GH dose Pre puberty (µg/kg/day)**	33.6 (2.2)<br>33.1 (29.8; 39.2)<br>n=23	58.9 (4.2)<br>60 (49.5; 63.4)<br>n=13	<.0001	-25.3 (-27.5; -23.2)	8.29
**Gain in $height_{SDS}$ GH start - Puberty onset**	1.43 (0.75)<br>1.55 (0.37; 2.87)<br>n=23	2.04 (0.65)<br>2.16 (0.86; 2.88)<br>n=13	0.019	-0.616 (-1.117; -0.114)	0.858

For continuous variables Mean (SD) / Median (Min; Max) / n= is presented.
For comparison between groups the Fisher´s Non Parametric Permutation Test was used for continuous variables.
The confidence interval for the mean difference between groups is based on Fishers non-parametric permutation test.
Effect size is absolute difference in mean / pooled SD.
GH, growth hormone; SDS, standard deviation score; MPH, mid-parental height; DiffMPH, difference in SD score between the height of the girl and the heights of her parents.

Suppl Table 2c Auxology at adult height for the **old** subgroup versus dose in the **PP** population versus the Swedish references [1,2,3,4]..

Variables	Dose 33 µg (n=23)	Dose 67 µg (n=13)	p-value	Difference between groups Mean (95% CI)	Effect Size
**At adult height**					
**Age (yrs)**	18.3 (1.5)<br>18.2 (15.9; 23.5)<br>n=23	17.2 (1.3)<br>17.6 (14.8; 18.9)<br>n=13	0.030	1.05 (0.10; 2.08)	0.741
**Adult height (cm)**	157.0 (4.1)<br>156.7 (145.7; 164)<br>n=23	159.4 (3.9)<br>159.7 (150.4; 165.5)<br>n=13	0.090	-2.44 (-5.34; 0.37)	0.605
**Height$_{SDS}$**	-1.75 (0.67)<br>-1.79 (-3.6; -0.59)<br>n=23	-1.35 (0.65)<br>-1.3 (-2.83; -0.34)<br>n=13	0.090	-0.400 (-0.877; 0.061)	0.604
**Diff MPH$_{SDS}$**	-1.47 (0.95)<br>-1.53 (-3.92; 0.03)<br>n=22	-1.42 (0.84)<br>-1.44 (-2.71; -0.18)<br>n=13	0.90	-0.043 (-0.686; 0.593)	0.048
**Gain in Height$_{SDS}$ Puberty onset - Adult height**	-0.349 (0.578)<br>-0.38 (-1.781; 1.321)<br>n=23	-0.573 (0.503)<br>-0.69 (-1.332; 0.24)<br>n=13	0.26	0.223 (-0.160; 0.612)	0.404
**Gain in height (cm) Puberty onset - Adult height**	7.76 (4.96)<br>8.2 (1.1; 20)<br>n=23	9.08 (5.14)<br>6.6 (3.8; 20.1)<br>n=13	0.45	-1.32 (-4.79; 2.26)	0.263
**Average GH dose$_{Total}$ (µg/kg/day)**	33.4 (1.5)<br>33.1 (31.2; 36.7)<br>n=23	59.2 (3.6)<br>60.2 (52.3; 63.4)<br>n=13	<.0001	-25.8 (-27.6; -24.1)	10.4
**Time on GH (yrs)**	5.37 (1.72)<br>6.01 (2.69; 8.29)<br>n=23	5.15 (1.24)<br>5.36 (2.62; 7.14)<br>n=13	0.69	0.223 (-0.893; 1.314)	0.142
**Duration puberty (yrs)**	3.32 (1.70)<br>3.43 (0.98; 8.15)<br>n=23	3.18 (0.95)<br>2.77 (1.99; 5.37)<br>n=13	0.82	0.138 (-0.842; 1.194)	0.093
**Gain in height$_{SDS}$ GH start - Adult height**	1.08 (0.84)<br>1.05 (-1.32; 2.39)<br>n=23	1.47 (0.62)<br>1.52 (-0.19; 2.23)<br>n=13	0.14	-0.393 (-0.941; 0.120)	0.513

For continuous variables Mean (SD) / Median (Min; Max) / n= is presented.
For comparison between groups the Fisher´s Non Parametric Permutation Test was used for continuous variables.
The confidence interval for the mean difference between groups is based on Fishers non-parametric permutation test.
Effect size is absolute difference in mean / pooled SD.
GH, growth hormone; SDS, standard deviation score; MPH, midparental height; DiffMPH, difference in SD score between the height of the girl and the heights of her parents.

Suppl Table 3a.Pre-treatment characteristics for **all girls** versus dose in the **ITT** population, versus Swedish references {1,2,3,4].

Variables	Dose 33 µg (n=67)	Dose 67 µg (n=65)	p-value	Difference between groups Mean (95% CI)	Effect Size
**Karyotype 45X**	38 (58.5%)	35 (53.8%)			
**Mosaic**	3 (4.6%)	2 (3.1%)			
**Other**	24 (36.9%)	28 (43.1%)	0.74		
**Missing**	2	0			
**At birth**					
**GA (weeks)**	39.0 (1.7)<br>39 (34; 42)<br>n=65	38.4 (2.0)<br>39 (33; 42)<br>n=62	0.10	0.551<br>(-0.097; 1.187)	0.301
**Length (SDS)**	-2.25 (1.42)<br>-2.09 (-5.49; 0.97)<br>n=67	-1.85 (1.18)<br>-1.73 (-4.48; 0.31)<br>n=64	0.082	-0.403<br>(-0.859; 0.054)	0.308
**Weight (SDS)**	-1.55 (1.25)<br>-1.3 (-5.11; 0.42)<br>n=67	-1.13 (1.17)<br>-1.04 (-3.92; 1.7)<br>n=65	0.049	-0.424<br>(-0.838; -0.001)	0.350
**Mother height (SDS)**	-0.247 (0.821)<br>-0.182 (-2.731; 1.3)<br>n=66	-0.008 (1.117)<br>-0.182 (-2.094; 2.633)<br>n=65	0.17	-0.239<br>(-0.575; 0.104)	0.244
**Father height (SDS)**	-0.092 (0.868)<br>-0.066 (-1.79; 1.818)<br>n=64	-0.050 (1.039)<br>0.144 (-3.326; 1.741)<br>n=65	0.80	-0.042<br>(-0.373; 0.291)	0.043
**MPH (SDS)**	-0.218 (0.820)<br>-0.238 (-2.046; 1.863)<br>n=64	-0.036 (1.022)<br>-0.16 (-3.356; 2.066)<br>n=65	0.27	-0.182<br>(-0.506; 0.141)	0.196
**DiffMPH (SDS)**	-2.08 (1.58)<br>-1.78 (-5.89; 1.48)<br>n=64	-1.83 (1.33)<br>-1.83 (-4.4; 0.93)<br>n=64	0.33	-0.247<br>(-0.756; 0.257)	0.169

For categorical variables n (%) is presented.
For continuous variables Mean (SD) / Median (Min; Max) / n= is presented.
For comparison between groups Chi Square Exact test was used for non-ordered categorical variables and the Fisher´s Non Parametric Permutation Test was used for continuous variables.
The confidence interval for the mean difference between groups is based on Fishers non-parametric permutation test.
Effect size is absolute difference in mean / pooled SD.
GA, gestational age; GH, growth hormone; SDS, standard deviation score; MPH, mid-parental height; DiffMPH, difference in SD score between the height of the girl and the heights of her parents.

Suppl Table 3b. Auxology from GH-start for **all girls** versus dose for the **ITT** population versus Swedish references {1,2,3,4}.

Variables	Dose 33 µg (n=67)	Dose 67 µg (n=65)	p-value	Difference between groups Mean (95% CI)	Effect Size
**At GH start**					
**Age (yrs)**	8.84 (3.48)<br>9 (3.04; 15.87)<br>n=67	7.29 (3.04)<br>6.96 (3.07; 14.77)<br>n=65	0.0088	1.55<br>(0.41; 2.67)	0.475
**Height$_{SDS}$**	-2.79 (0.77)<br>-2.78 (-4.43; -1.13)<br>n=67	-2.79 (0.67)<br>-2.79 (-5.39; -1.44)<br>n=65	0.96	0.007<br>(-0.240; 0.250)	0.009
**Diff MPH$_{SDS}$**	-2.54 (0.86)<br>-2.4 (-4.23; -0.56)<br>n=64	-2.76 (0.87)<br>-2.73 (-4.5; -1.08)<br>n=65	0.15	0.220<br>(-0.081; 0.520)	0.254
**+ 1 yr All prepubertal**					
**Average GH dose (µg/kg/day)**	34.8 (4.8)<br>34.2 (25.5; 61)<br>n=67	54.7 (7.2)<br>56.1 (28; 71.4)<br>n=65	<.0001	-19.9<br>(-22.0; -17.8)	3.27
**Height$_{SDS}$**	-2.31 (0.75)<br>-2.29 (-3.87; -0.66)<br>n=61	-1.88 (0.68)<br>-1.8 (-4.35; -0.41)<br>n=65	0.0010	-0.427<br>(-0.676; -0.173)	0.600
**At puberty start**					
**Age (yrs)**	14.9 (1.4)<br>15 (11.2; 18)<br>n=67	13.4 (1.6)<br>13.7 (9.1; 16.8)<br>n=65	<.0001	1.58<br>(1.07; 2.10)	1.07
**Height$_{SDS}$**	-1.29 (0.83)<br>-1.36 (-3.43; 0.27)<br>n=67	-0.514 (0.897)<br>-0.41 (-3.022; 1.258)<br>n=65	<.0001	-0.772<br>(-1.069; -0.477)	0.894
**Diff MPH$_{SDS}$**	-1.07 (1.01)<br>-1.22 (-3.39; 0.8)<br>n=64	-0.478 (1.008)<br>-0.504 (-2.412; 1.829)<br>n=65	0.0007	-0.592<br>(-0.944; -0.243)	0.588
**Average GH dose Pre puberty (µg/kg/day)**	34.4 (2.9)<br>33.8 (29.8; 46.7)<br>n=65	57.2 (5.9)<br>58.2 (37.7; 68.9)<br>n=65	<.0001	-22.8<br>(-24.4; -21.2)	4.89
**Gain in height$_{SDS}$ GH start - Puberty onset**	1.50 (0.80)<br>1.54 (0; 3.43)<br>n=67	2.28 (0.73)<br>2.26 (0.81; 4.38)<br>n=65	<.0001	-0.778<br>(-1.041; -0.516)	1.02

For continuous variables Mean (SD) / Median (Min; Max) / n= is presented.
For comparison between groups the Fisher´s Non Parametric Permutation Test was used for continuous variables. The confidence interval for the mean difference between groups is based on Fishers non-parametric permutation test.
Effect size is absolute difference in mean / pooled SD.
GH, growth hormone; SDS, standard deviation score; MPH, mid-parental height; DiffMPH, difference in SD score between the height of the girl and the heights of her parents.

Suppl Table 3c Auxology at adult height for **all girls** versus dose in the **ITT** population versus the Swedish references {1,2,3,4}

Variables	Dose 33 µg (n=67)	Dose 67 µg (n=65)	p-value	Difference between groups Mean (95% CI)	Effect Size
**At adult height**					
**Age (yrs)**	17.9 (1.7)<br>17.5 (15.6; 25.9)<br>n=67	16.4 (1.2)<br>16.4 (13.4; 19.8)<br>n=65	<.0001	1.44<br>(0.92; 1.96)	0.948
**Adult height (cm)**	155.1 (5.4)<br>155.7 (143.7; 169.2)<br>n=67	158.0 (5.4)<br>158.1 (145.1; 168.3)<br>n=65	0.0026	-2.82<br>(-4.66; -1.00)	0.524
**$Height_{SDS}$**	-2.05 (0.89)<br>-1.96 (-3.93; 0.27)<br>n=67	-1.58 (0.88)<br>-1.56 (-3.7; 0.12)<br>n=65	0.0026	-0.464<br>(-0.767; -0.165)	0.524
**Diff MPH_{SDS}**	-1.84 (0.98)<br>-1.84 (-4.53; 0.03)<br>n=64	-1.55 (0.89)<br>-1.64 (-4.07; 0.49)<br>n=65	0.071	-0.299<br>(-0.625; 0.028)	0.320
**Gain in $Height_{SDS}$<br>Puberty onset - Adult height**	-0.761 (0.665)<br>-0.79 (-1.878; 1.626)<br>n=67	-1.07 (0.65)<br>-1.11 (-2.63; 0.66)<br>n=65	0.0096	0.308<br>(0.082; 0.534)	0.468
**Gain in height (cm)<br>Puberty onset - Adult height**	5.23 (4.72)<br>3.7 (-0.2; 21.1)<br>n=67	8.10 (5.95)<br>6.2 (0; 26.8)<br>n=65	0.0033	-2.87<br>(-4.74; -1.00)	0.536
**Average GH $dose_{Total}$<br>(µg/kg/day)**	34.7 (3.6)<br>33.7 (30.3; 48.1)<br>n=67	55.8 (6.4)<br>56.4 (37.3; 69.1)<br>n=65	<.0001	-21.1<br>(-22.8; -19.3)	4.08
**Time on GH (yrs)**	7.66 (3.07)<br>7.26 (1.99; 13.02)<br>n=67	8.22 (2.62)<br>8.03 (2.62; 13.87)<br>n=65	0.27	-0.556<br>(-1.546; 0.436)	0.195
**Duration puberty (yrs)**	2.94 (1.55)<br>2.7 (0.61; 8.7)<br>n=67	3.08 (1.44)<br>3.05 (0; 9.26)<br>n=65	0.58	-0.144<br>(-0.661; 0.378)	0.096
**Gain in $height_{SDS}$<br>GH start - Adult height**	0.741 (0.825)<br>0.694 (-1.32; 2.664)<br>n=67	1.21 (0.71)<br>1.24 (-0.52; 3.27)<br>n=65	0.0006	-0.470<br>(-0.740; -0.206)	0.609

For continuous variables Mean (SD) / Median (Min; Max) / n= is presented.
For comparison between groups the Fisher´s Non Parametric Permutation Test was used for continuous variables.
The confidence interval for the mean difference between groups is based on Fishers non-parametric permutation test.
Effect size is absolute difference in mean / pooled SD.
GH, growth hormone; SDS, standard deviation score; MPH, midparental height; DiffMPH, difference in SD score between the height of the girl and the heights of her parents.

Suppl Table 4a. Pre-treatment characteristics for **all GH** versus age group at GH-start in **ITT** population versus the Swedish references {1,2,3,4}.

Variables	GH-start 3- <9 (n=79)	GH-start 9- (n=53)	p-value	Difference between groups Mean (95% CI)	Effect Size
**Karyotype 45X**	50 (64.9%)	23 (43.4%)			
**Mosaic**	3 (3.9%)	2 (3.8%)			
**Other**	24 (31.2%)	28 (52.8%)	0.038		
**Missing**	2	0			
**At birth**					
**GA (weeks)**	38.5 (2.0)<br>39 (33; 42)<br>n=77	39.0 (1.6)<br>39 (34; 42)<br>n=50	0.19	-0.454<br>(-1.115; 0.207)	0.247
**Length (SDS)**	-2.14 (1.31)<br>-2.09 (-5; 0.9)<br>n=79	-1.92 (1.33)<br>-1.73 (-5.49; 0.97)<br>n=52	0.36	-0.220<br>(-0.685; 0.251)	0.167
**Weight (SDS)**	-1.33 (1.32)<br>-1.23 (-5.11; 1.7)<br>n=79	-1.36 (1.09)<br>-1.2 (-4.73; 1.41)<br>n=53	0.88	0.032<br>(-0.400; 0.469)	0.026
**Mother height (SDS)**	-0.092 (1.103)<br>-0.182 (-2.731; 2.633)<br>n=79	-0.183 (0.771)<br>-0.182 (-1.775; 1.73)<br>n=52	0.60	0.091<br>(-0.252; 0.440)	0.092
**Father height (SDS)**	-0.169 (1.014)<br>-0.03 (-3.326; 1.741)<br>n=79	0.084 (0.838)<br>0.052 (-1.79; 1.818)<br>n=50	0.15	-0.253<br>(-0.590; 0.088)	0.266
**MPH (SDS)**	-0.162 (1.021)<br>-0.238 (-3.356; 2.066)<br>n=79	-0.069 (0.766)<br>-0.038 (-1.547; 1.775)<br>n=50	0.58	-0.093<br>(-0.429; 0.238)	0.100
**DiffMPH (SDS)**	-1.98 (1.50)<br>-2.02 (-5.79; 1.48)<br>n=79	-1.92 (1.39)<br>-1.71 (-5.89; 0.96)<br>n=49	0.85	-0.053<br>(-0.579; 0.473)	0.037

For categorical variables n (%) is presented.
For continuous variables Mean (SD) / Median (Min; Max) / n= is presented.
For comparison between groups Chi Square Exact test was used for non-ordered categorical variables and the Fisher´s Non Parametric Permutation Test was used for continuous variables.
The confidence interval for the mean difference between groups is based on Fishers non-parametric permutation test.
Effect size is absolute difference in mean / pooled SD.
GA, gestational age; GH, growth hormone; SDS, standard deviation score; MPH, mid-parental height; DiffMPH, difference in SD score between the height of the girl and the heights of her parents.

Suppl Table 4b. Auxology from GH-start for **all GH** versus age group at GH-start in the **ITT** population versus the Swedish references {1,2,3,4].

Variables	GH-start 3- <9 (n=79)	GH-start 9- (n=53)	p-value	Difference between groups Mean (95% CI)	Effect Size
**At GH start**					
**Age (yrs)**	5.74 (1.64)<br>5.52 (3.04; 8.9)<br>n=79	11.6 (1.9)<br>10.9 (9; 15.9)<br>n=53	<.0001	-5.82<br>(-6.43; -5.20)	3.34
**Height$_{SDS}$**	-2.78 (0.74)<br>-2.78 (-5.39; -1.13)<br>n=79	-2.81 (0.69)<br>-2.84 (-4.43; -1.36)<br>n=53	0.85	0.026<br>(-0.228; 0.274)	0.036
**Diff MPH$_{SDS}$**	-2.62 (0.92)<br>-2.59 (-4.5; -0.56)<br>n=79	-2.70 (0.78)<br>-2.58 (-4.01; -0.86)<br>n=50	0.62	0.080<br>(-0.234; 0.394)	0.092
**+ 1 yr All prepubertal**					
**Average GH dose$_{Year\ 1}$ (µg/kg/day)**	46.3 (11.3)<br>48.2 (25.5; 66.4)<br>n=79	41.9 (11.8)<br>35.8 (26.7; 71.4)<br>n=53	0.031	4.45<br>(0.43; 8.49)	0.386
**Height$_{SDS)}$**	-2.08 (0.74)<br>-2.07 (-4.35; -0.51)<br>n=79	-2.10 (0.75)<br>-2.1 (-3.87; -0.41)<br>n=47	0.86	0.024<br>(-0.252; 0.294)	0.032
**At puberty start**					
**Age (yrs)**	13.7 (1.6)<br>13.9 (9.1; 16.9)<br>n=79	14.8 (1.7)<br>15.2 (11.2; 18)<br>n=53	0.0003	-1.06<br>(-1.62; -0.50)	0.665
**Height$_{SDS}$**	-0.705 (0.987)<br>-0.649 (-3.184; 1.258)<br>n=79	-1.20 (0.79)<br>-1.17 (-3.43; 0.3)<br>n=53	0.0029	0.499<br>(0.175; 0.821)	0.546
**Diff MPH$_{SDS}$**	-0.543 (1.095)<br>-0.477 (-3.394; 1.829)<br>n=79	-1.13 (0.86)<br>-1.37 (-3.02; 0.68)<br>n=50	0.0014	0.590<br>(0.230; 0.957)	0.584
**Average GH dose Pre puberty (µg/kg/day)**	47.6 (11.9)<br>52 (30.3; 65.4)<br>n=79	43.0 (12.6)<br>35.7 (29.8; 68.9)<br>n=51	0.039	4.57<br>(0.23; 8.93)	0.375
**Gain in Height$_{SDS}$ GH start - Puberty onset**	2.07 (0.78)<br>2.15 (0.48; 4.38)<br>n=79	1.60 (0.89)<br>1.71 (0; 3.43)<br>n=53	0.0026	0.472<br>(0.179; 0.766)	0.572

For continuous variables Mean (SD) / Median (Min; Max) / n= is presented.
For comparison between groups the Fisher´s Non Parametric Permutation Test was used for continuous variables.
The confidence interval for the mean difference between groups is based on Fishers non-parametric permutation test.
Effect size is absolute difference in mean / pooled SD.
GH, growth hormone; SDS, standard deviation score; MPH, mid-parental height; DiffMPH, difference in SD score between the height of the girl and the heights of her parents.

Suppl Table 4c. Auxology at adult height for **all GH** versus age group at GH-start in the **ITT** population versus the Swedish references {1,2,3,4].

Variables	GH-start 3- <9 (n=79)	GH-start 9- (n=53)	p-value	Difference between groups Mean (95% CI)	Effect Size
**At adult height**					
**Age (yrs)**	16.6 (1.3)<br>16.5 (13.4; 21.4)<br>n=79	18.0 (1.8)<br>17.8 (14.8; 25.9)<br>n=53	<.0001	-1.41<br>(-1.93; -0.86)	0.917
**Adult height (cm)**	155.7 (5.8)<br>155.7 (143.7; 168.3)<br>n=79	157.7 (4.9)<br>158.1 (144; 169.2)<br>n=53	0.042	-2.00<br>(-3.93; -0.09)	0.364
**Height$_{SDS}$**	-1.95 (0.96)<br>-1.96 (-3.93; 0.12)<br>n=79	-1.62 (0.80)<br>-1.56 (-3.88; 0.27)<br>n=53	0.042	-0.328<br>(-0.647; -0.015)	0.365
**Diff MPH$_{SDS}$**	-1.79 (0.97)<br>-1.77 (-4.53; 0.49)<br>n=79	-1.55 (0.89)<br>-1.72 (-3.92; 0.03)<br>n=50	0.16	-0.240<br>(-0.577; 0.100)	0.255
**Gain in height$_{SDS}$**<br>**Puberty onset - Adult height**	-1.24 (0.50)<br>-1.31 (-2.63; 0)<br>n=79	-0.417 (0.598)<br>-0.49 (-1.781; 1.626)<br>n=53	<.0001	-0.827<br>(-1.017; -0.638)	1.54
**Gain in height (cm)**<br>**Puberty onset - Adult height**	5.82 (5.08)<br>4.6 (-0.2; 23.3)<br>n=79	7.87 (6.00)<br>5.6 (0.9; 26.8)<br>n=53	0.039	-2.05<br>(-3.96; -0.11)	0.375
**Average GH dose$_{Total}$**<br>**(µg/kg/day)**	46.6 (11.3)<br>48.1 (30.3; 65.9)<br>n=79	42.9 (12.2)<br>36.1 (30.4; 69.1)<br>n=53	0.071	3.77<br>(-0.32; 7.85)	0.324
**Time on GH (yrs)**	9.75 (1.97)<br>9.9 (5.3; 13.87)<br>n=79	5.22 (1.50)<br>5.44 (1.99; 8.29)<br>n=53	<.0001	4.53<br>(3.90; 5.16)	2.52
**Duration puberty (yrs)**	2.87 (1.40)<br>2.78 (0; 9.26)<br>n=79	3.22 (1.61)<br>3.21 (0.61; 8.7)<br>n=53	0.20	-0.344<br>(-0.858; 0.183)	0.231
**Gain in height$_{SDS}$**<br>**GH start - Adult height**	0.830 (0.759)<br>0.817 (-0.694; 3.269)<br>n=79	1.18 (0.83)<br>1.48 (-1.32; 2.66)<br>n=53	0.014	-0.354<br>(-0.630; -0.076)	0.450

For continuous variables Mean (SD) / Median (Min; Max) / n= is presented.
For comparison between groups the Fisher´s Non Parametric Permutation Test was used for continuous variables.
The confidence interval for the mean difference between groups is based on Fishers non-parametric permutation test.
Effect size is absolute difference in mean / pooled SD.
AH, adult height; GH, growth hormone; ns, not significant; SDS, standard deviation score; MPH, midparental height; DiffMPH, difference in SD score between the height of the girl and the heights of her parents.

Suppl Table 5. Growth from birth to adult height for **all GH** versus age group at GH-start and dose in the **PP** population; expressed in SDS versus Turner reference [5,6]

	Group									
	GH-start 3- <9					GH-start 9-				
**Variables**	**Dose 33 µg**	**Dose 67 µg**	**p-value**	**Difference between groups Mean (95% CI)**	**Effect Size**	**Dose 33 µg**	**Dose 67 µg**	**p-value**	**Difference between groups Mean (95% CI)**	**Effect Size**
	n=25	n=28				n=23	n=13			
**At birth**										
**Length SDS**	-0.520 (1.007)<br>-0.24<br>(-2.64; 0.96)<br>n=25	-0.261 (0.921)<br>-0.04<br>(-2.24; 1.76)<br>n=28	0.36	-0.259<br>(-0.786; 0.282)	0.269	-0.110 (0.839)<br>-0.24<br>(-2.24; 1.36)<br>n=23	-0.307 (1.007)<br>-0.04<br>(-2.24; 0.96)<br>n=12	0.57	0.197<br>(-0.457; 0.844)	0.219
**At GH start**										
**Height SDS**	-0.272 (0.783)<br>-0.264<br>(-1.545; 1.479)<br>n=25	-0.323 (0.978)<br>-0.28<br>(-3.145; 1.449)<br>n=28	0.83	0.051<br>(-0.433; 0.555)	0.058	0.169 (0.950)<br>0.176<br>(-1.804; 2.003)<br>n=23	0.157 (0.695)<br>-0.023<br>(-0.95; 1.933)<br>n=13	0.96	0.012<br>(-0.586; 0.619)	0.014
**+ 1 year All prepubertal**										
**Height$_{SDS}$**	0.431 (0.803)<br>0.412<br>(-0.976; 2.159)<br>n=25	0.749 (0.979)<br>0.884<br>(-1.853; 2.737)<br>n=28	0.21	-0.318<br>(-0.810; 0.186)	0.353	0.848 (0.960)<br>0.708<br>(-1.062; 2.609)<br>n=20	1.45 (0.70)<br>1.43<br>(0.39; 3.05)<br>n=13	0.067	-0.598<br>(-1.231; 0.047)	0.687
**At puberty start**										
**Height SDS**	2.41 (0.82)<br>2.43<br>(0.75; 3.77)<br>n=25	2.89 (1.19)<br>2.97<br>(-0.1; 4.92)<br>n=28	0.10	-0.477<br>(-1.048; 0.098)	0.462	1.96 (0.58)<br>1.85<br>(1.08; 3.02)<br>n=23	2.61 (0.70)<br>2.86<br>(1.37; 3.42)<br>n=13	0.0052	-0.649<br>(-1.098; -0.200)	1.04
**At adult height**										
**Height SDS**	1.94 (0.93)<br>1.84<br>(0.11; 3.62)<br>n=25	2.73 (1.09)<br>2.88<br>(0.77; 4.86)<br>n=28	0.0085	-0.793<br>(-1.362; -0.221)	0.779	2.11 (0.50)<br>2.22<br>(0.92; 2.9)<br>n=23	2.78 (0.52)<br>3<br>(1.78; 3.41)<br>n=13	0.0005	-0.675<br>(-1.043; -0.315)	1.32
**Gain in height**										
**Gain in height SDS**<br>**GH start - Puberty onset**	2.68 (0.67)<br>2.57<br>(1.54; 4.48)<br>n=25	3.21 (0.87)<br>3.14<br>(1.74; 5.3)<br>n=28	0.018	-0.528<br>(-0.955; -0.094)	0.675	1.79 (0.96)<br>1.97<br>(0.43; 3.55)<br>n=23	2.45 (0.80)<br>2.64<br>(1.08; 3.42)<br>n=13	0.041	-0.661<br>(-1.291; -0.030)	0.732
**Gain in Height SDS**<br>**Puberty onset - Adult height**	-0.474 (0.297)<br>-0.479<br>(-1.202; -0.109)<br>n=25	-0.158 (0.463)<br>-0.196<br>(-1.147; 0.867)<br>n=28	0.0045	-0.316<br>(-0.533; -0.099)	0.802	0.150 (0.412)<br>0.121<br>-0.59; 1.242)<br>n=23	0.176 (0.379)<br>0.248<br>(-0.293; 1.002)<br>n=13	0.84	-0.026<br>(-0.302; 0.258)	0.064
**Gain in height SDS**<br>**GH start - Adult height**	2.21 (0.73)<br>2.3<br>(0.69; 3.44)<br>n=25	3.05 (0.78)<br>2.99<br>(1.47; 4.78)<br>n=28	0.0002	-0.844<br>1.260; -0.428)	1.12	1.94 (0.90)<br>2.1<br>(0.1; 3.58)<br>n=23	2.63 (0.65)<br>2.57<br>(1.41; 3.63)<br>n=13	0.020	-0.687<br>(-1.263; -0.117)	0.835

For continuous variables Mean (SD) / Median (Min; Max) / n= is presented.
For comparison between groups the Fisher´s Non Parametric Permutation Test was used for continuous variables.
The confidence interval for the mean difference between groups is based on Fishers non-parametric permutation test.
Effect size is absolute difference in mean / pooled SD.
AH, adult height; GH, growth hormone; SDS, standard deviation score.

Suppl Table 6a. Pre-treatment characteristics for **all girls** versus dose in the **PP** population, versus the Swedish references {I,2,3,4]

Variables	Dose 33 µg (n=48)	Dose 67 µg (n=41)	p-value	Difference between groups Mean (95% CI)	Effect Size
**Karyotype 45X**	27 (56.3%)	23 (56.1%)			
**Mosaic**	2 (4.2%)	1 (2.4%)			
**Other**	19 (39.6%)	17 (41.5%)	1.00		
**Missing**	0	0			
**At birth**	39.0 (1.5)<br>39 (36; 42)<br>n=46	38.7 (1.8)<br>39 (35; 42)<br>n=38	0.50	0.272 (-0.471; 1.000)	0.162
**GA (weeks)**	-2.30 (1.56)<br>-2.21 (-5.49; 0.97)<br>n=48	-2.09 (1.14)<br>-1.94 (-4.48; 0.23)<br>n=40	0.48	-0.215 (-0.812; 0.381)	0.155
**Length (SDS)**	-1.59 (1.34)<br>-1.35 (-5.11; 0.42)<br>n=48	-1.28 (1.16)<br>-0.98 (-3.92; 1.41)<br>n=41	0.26	-0.311 (-0.845; 0.225)	0.247
**Weight (SDS)**	-0.265 (0.774)<br>-0.261 (-1.504; 1.093)<br>n=48	-0.032 (1.145)<br>-0.182 (-2.078; 2.333)<br>n=41	0.25	-0.233 (-0.646; 0.175)	0.242
**Mother height (SDS)**	-0.160 (0.884)<br>-0.255 (-1.79; 1.818)<br>n=47	-0.148 (1.112)<br>-0.101 (-3.326; 1.588)<br>n=41	0.95	-0.012 (-0.434; 0.407)	0.012
**Father height (SDS)**	-0.282 (0.765)<br>-0.444 (-2.046; 1.185)<br>n=47	-0.112 (1.114)<br>-0.238 (-3.356; 2.066)<br>n=41	0.39	-0.170 (-0.577; 0.226)	0.181
**MPH (SDS)**	-2.03 (1.70)<br>-1.71 (-5.89; 1.48)<br>n=47	-2.01 (1.28)<br>-2.03 (-4.4; 0.9)<br>n=40	0.96	-0.016 (-0.676; 0.628)	0.011

For categorical variables n (%) is presented.
For continuous variables Mean (SD) / Median (Min; Max) / n= is presented.
For comparison between groups Chi Square Exact test was used for non-ordered categorical variables and the Fisher´s Non Parametric Permutation Test was used for continuous variables.
The confidence interval for the mean difference between groups is based on Fishers non-parametric permutation test.
Effect size is absolute difference in mean / pooled SD.
GA, gestational age; GH, growth hormone; SDS, standard deviation score; MPH, mid-parental height; DiffMPH, difference in SD score between the height of the girl and the heights of her parents.

Suppl Table 6b. Auxology from GH-start for **all girls** versus dose in the **PP** population versus the Swedish references [1,2,3,4].

Variables	Dose 33 µg (n=48)	Dose 67 µg (n=41)	p-value	Difference between groups Mean (95% CI)	Effect Size
**At GH start**					
**Age (yrs)**	8.67 (3.43)<br>8.59 (3.04; 15.87)<br>n=48	7.59 (3.15)<br>7.17 (3.15; 14.77)<br>n=41	0.12	1.07 (-0.34; 2.45)	0.325
**Height$_{SDS}$**	-2.81 (0.74)<br>-2.81 (-4.43; -1.13)<br>n=48	-2.84 (0.74)<br>-2.93 (-5.39; -1.44)<br>n=41	0.82	0.033 (-0.278; 0.342)	0.045
**Diff MPH$_{SDS}$**	-2.53 (0.85)<br>-2.38 (-4.12; -0.56)<br>n=47	-2.73 (0.92)<br>-2.68 (-4.5; -1.08)<br>n=41	0.26	0.207 (-0.163; 0.581)	0.235
**+ 1 yr All prepubertal**					
**Average GH dose (µg/kg/day)**	34.3 (3.7)<br>34.5 (25.5; 41.6)<br>n=48	55.0 (5.5)<br>55.5 (39.4; 66.4)<br>n=41	<.0001	-20.8 (-22.7; -18.8)	4.48
**Height$_{SDS}$**	-2.27 (0.76)<br>-2.29 (-3.87; -0.66)<br>n=45	-1.92 (0.74)<br>-1.8 (-4.35; -0.41)<br>n=41	0.034	-0.348 (-0.669; -0.028)	0.464
**At puberty start**					
**Age (yrs)**	14.8 (1.4)<br>14.8 (11.2; 16.9)<br>n=48	13.2 (1.5)<br>13.3 (10.6; 16.6)<br>n=41	<.0001	1.58 (0.97; 2.18)	1.11
**Height$_{SDS}$**	-1.19 (0.69)<br>-1.17 (-2.65; 0.27)<br>n=48	-0.596 (0.915)<br>-0.417 (-3.022; 1.258)<br>n=41	0.0012	-0.590 (-0.929; -0.252)	0.737
**Diff MPH$_{SDS}$**	-0.904 (0.876)<br>-1.045 (-2.791; 0.798)<br>n=47	-0.484 (0.969)<br>-0.364 (-2.412; 1.794)<br>n=41	0.036	-0.420 (-0.805; -0.028)	0.456
**Average GH dose Pre puberty (µg/kg/day)**	33.8 (1.9)<br>33.5 (29.8; 39.2)<br>n=48	58.8 (4.2)<br>59.6 (46.7; 65.4)<br>n=41	<.0001	-25.0 (-26.4; -23.7)	7.80
**Gain in height$_{SDS}$ GH start - Puberty onset**	1.62 (0.71)<br>1.68 (0.37; 3.4)<br>n=48	2.25 (0.75)<br>2.21 (0.81; 4.38)<br>n=41	0.0002	-0.623 (-0.930; -0.311)	0.854

For continuous variables Mean (SD) / Median (Min; Max) / n= is presented.
For comparison between groups the Fisher´s Non Parametric Permutation Test was used for continuous variables.
The confidence interval for the mean difference between groups is based on Fishers non-parametric permutation test.
Effect size is absolute difference in mean / pooled SD.
GH, growth hormone; SDS, standard deviation score; MPH, mid-parental height; DiffMPH, difference in SD score between the height of the girl and the heights of her parents.

Suppl Table 6c Auxology at adult height for **all girls** versus dose in the **PP** population versus the Swedish references [1,2,3,4]..

Variables	Dose 33 µg (n=48)	Dose 67 µg (n=41)	p-value	Difference between groups Mean (95% CI)	Effect Size
**At adult height**					
**Age (yrs)**	17.8 (1.4)<br>17.5 (15.6; 23.5)<br>n=48	16.6 (1.3)<br>16.4 (14.7; 19.8)<br>n=41	<.0001	1.21 (0.64; 1.79)	0.894
**Adult height (cm)**	155.6 (4.7)<br>155.8 (145.7; 165.4)<br>n=48	157.8 (5.9)<br>158.1 (145.1; 168.3)<br>n=41	0.058	-2.18 (-4.42; 0.07)	0.411
**Height$_{SDS}$**	-1.97 (0.77)<br>-1.94 (-3.6; -0.36)<br>n=48	-1.61 (0.97)<br>-1.56 (-3.7; 0.12)<br>n=41	0.058	-0.358 (-0.727; 0.012)	0.411
**Diff MPH$_{SDS}$**	-1.69 (0.89)<br>-1.77 (-3.92; 0.03)<br>n=47	-1.50 (0.96)<br>-1.46 (-4.07; 0.49)<br>n=41	0.35	-0.188 (-0.578; 0.204)	0.204
**Gain in Height$_{SDS}$ Puberty onset - Adult height**	-0.786 (0.642)<br>-0.773 (-1.878; 1.321)<br>n=48	-1.02 (0.57)<br>-0.99 (-2.08; 0.24)<br>n=41	0.074	0.232 (-0.023; 0.489)	0.382
**Gain in height (cm) Puberty onset - Adult height**	5.55 (4.49)<br>4 (-0.2; 20)<br>n=48	9.09 (5.28)<br>7.6 (0.8; 20.5)<br>n=41	0.0010	-3.54 (-5.60; -1.48)	0.728
**Average GH dose$_{Total}$ (µg/kg/day)**	33.7 (1.7)<br>33.4 (31; 38.3)<br>n=48	58.3 (4.1)<br>58.6 (46.6; 65.9)<br>n=41	<.0001	-24.6 (-25.9; -23.3)	8.01
**Time on GH (yrs)**	7.98 (3.01)<br>8.32 (2.69; 12.61)<br>n=48	8.07 (2.70)<br>8.03 (2.62; 13.87)<br>n=41	0.88	-0.095 (-1.287; 1.117)	0.033
**Duration puberty (yrs)**	3.00 (1.43)<br>2.94 (0.98; 8.15)<br>n=48	3.37 (1.45)<br>3.21 (1.02; 9.26)<br>n=41	0.25	-0.361 (-0.967; 0.256)	0.251
**Gain in height$_{SDS}$ GH start - Adult height**	0.839 (0.785)<br>0.821 (-1.32; 2.388)<br>n=48	1.23 (0.75)<br>1.24 (-0.52; 3.27)<br>n=41	0.020	-0.391 (-0.714; -0.064)	0.508

For continuous variables Mean (SD) / Median (Min; Max) / n= is presented.
For comparison between groups the Fisher´s Non Parametric Permutation Test was used for continuous variables.
The confidence interval for the mean difference between groups is based on Fishers non-parametric permutation test.
Effect size is absolute difference in mean / pooled SD.
GH, growth hormone; SDS, standard deviation score; MPH, midparental height; DiffMPH, difference in SD score between the height of the girl and the heights of her parents.

Suppl Table 7a. Pre-treatment characteristics for **all GH** versus age group at GH-start in the PP population versus the Swedish reference {1,2,3,4}.

Variables	GH-start 3- <9 (n=53)	GH-start 9- (n=36)	p-value	Difference between groups Mean (95% CI)	Effect Size
**Karyotype 45X**	35 (66.0%)	15 (41.7%)			
**Mosaic**	2 (3.8%)	1 (2.8%)			
**Other**	16 (30.2%)	20 (55.6%)	0.045		
**Missing**					
**At birth**					
**GA (weeks)**	38.7 (1.8)<br>39 (35; 42)<br>n=51	39.1 (1.5)<br>39 (36; 42)<br>n=33	0.35	-0.374 (-1.118; 0.364)	0.223
**Length (SDS)**	-2.27 (1.35)<br>-2.09 (-5; 0.9)<br>n=53	-2.11 (1.44)<br>-1.93 (-5.49; 0.97)<br>n=35	0.63	-0.150 (-0.748; 0.464)	0.108
**Weight (SDS)**	-1.38 (1.33)<br>-1.16 (-5.11; 0.37)<br>n=53	-1.54 (1.18)<br>-1.35 (-4.73; 1.41)<br>n=36	0.57	0.155 (-0.389; 0.697)	0.122
**Mother height (SDS)**	-0.122 (1.094)<br>-0.261 (-2.078; 2.333)<br>n=53	-0.211 (0.744)<br>-0.182 (-1.775; 1.571)<br>n=36	0.67	0.089 (-0.329; 0.510)	0.092
**Father height (SDS)**	-0.268 (1.070)<br>-0.255 (-3.326; 1.434)<br>n=53	0.017 (0.844)<br>-0.101 (-1.713; 1.818)<br>n=35	0.19	-0.284 (-0.711; 0.142)	0.288
**MPH (SDS)**	-0.242 (1.065)<br>-0.429 (-3.356; 2.066)<br>n=53	-0.144 (0.727)<br>-0.077 (-1.547; 1.058)<br>n=35	0.63	-0.098 (-0.507; 0.308)	0.104
**DiffMPH (SDS)**	-2.02 (1.53)<br>-2.03 (-5.79; 1.48)<br>n=53	-2.02 (1.51)<br>-1.71 (-5.89; 0.96)<br>n=34	0.99	-0.006 (-0.662; 0.655)	0.004

For categorical variables n (%) is presented.
For continuous variables Mean (SD) / Median (Min; Max) / n= is presented.
For comparison between groups Chi Square Exact test was used for non-ordered categorical variables and the Fisher´s Non Parametric Permutation Test was used for continuous variables.
The confidence interval for then mean difference between groups is based on Fishers non-parametric permutation test.
Effect size is absolute difference in mean / pooled SD.
GA, gestational age; GH, growth hormone; SDS, standard deviation score; MPH, mid-parental height; DiffMPH, difference in SD score between the height of the girl and the heights of her parents.

Suppl Table 7b. Auxology from GH-start for **all GH** versus age group at GH-start in the **PP** population versus the Swedish references [1,2,3,4].

Variables	GH-start 3- <9 (n=53)	GH-start 9- (n=36)	p-value	Difference between groups Mean (95% CI)	Effect Size
**At GH start**					
**Age (yrs)**	5.82 (1.61)<br>5.55 (3.04; 8.89)<br>n=53	11.6 (1.8)<br>11.1 (9; 15.9)<br>n=36	<.0001	-5.81 (-6.53; -5.08)	3.42
**$Height_{SDS}$**	-2.83 (0.77)<br>-2.82 (-5.39; -1.13)<br>n=53	-2.82 (0.69)<br>-2.88 (-4.43; -1.36)<br>n=36	0.97	-0.006 (-0.320; 0.310)	0.008
**Diff MPH_{SDS}**	-2.59 (0.93)<br>-2.4 (-4.5; -0.56)<br>n=53	-2.67 (0.81)<br>-2.5 (-4.01; -0.86)<br>n=35	0.64	0.088 (-0.290; 0.468)	0.099
**+ 1 yr All prepubertal**					
**Average GH $dose_{Year\ 1}$ (μg/kg/day)**	45.3 (10.9)<br>43.6 (25.5; 66.4)<br>n=53	41.7 (11.8)<br>35.8 (26.7; 62.5)<br>n=36	0.14	3.55 (-1.28; 8.35)	0.314
**$Height_{SDS)}$**	-2.13 (0.77)<br>-2.16 (-4.35; -0.51)<br>n=53	-2.07 (0.76)<br>-2 (-3.87; -0.41)<br>n=33	0.72	-0.065 (-0.403; 0.270)	0.084
**At puberty start**					
**Age (yrs)**	13.6 (1.5)<br>13.7 (10.6; 16.9)<br>n=53	14.6 (1.6)<br>15 (11.2; 16.8)<br>n=36	0.0038	-0.996 (-1.680; -0.336)	0.643
**$Height_{SDS}$**	-0.738 (0.937)<br>-0.696 (-3.022; 1.258)<br>n=53	-1.17 (0.62)<br>-1.17 (-2.28; -0.07)<br>n=36	0.015	0.436 (0.088; 0.790)	0.528
**Diff MPH_{SDS}**	-0.496 (0.993)<br>-0.459 (-2.791; 1.794)<br>n=53	-1.03 (0.76)<br>-1.29 (-2.14; 0.67)<br>n=35	0.0074	0.535 (0.145; 0.930)	0.590
**Average GH dose Pre puberty (μg/kg/day)**	47.1 (12.9)<br>52 (30.8; 65.4)<br>n=53	42.7 (12.7)<br>35.2 (29.8; 63.4)<br>n=36	0.12	4.35 (-1.06; 9.84)	0.339
**Gain in $Height_{SDS}$ GH start - Puberty onset**	2.09 (0.76)<br>2.09 (0.61; 4.38)<br>n=53	1.65 (0.77)<br>1.73 (0.37; 2.88)<br>n=36	0.0070	0.442 (0.117; 0.767)	0.579

For continuous variables Mean (SD) / Median (Min; Max) / n= is presented.
For comparison between groups the Fisher´s Non Parametric Permutation Test was used for continuous variables.
The confidence interval for the mean difference between groups is based on Fishers non-parametric permutation test.
Effect size is absolute difference in mean / pooled SD.
GH, growth hormone; SDS, standard deviation score; MPH, mid-parental height; DiffMPH, difference in SD score between the height of the girl and the heights of her parents.

Suppl Table 7c. Auxology at adult height for **all GH** versus age group at GH-start in the **PP** population versus the Swedish references [1,2,3,4].

Variables	GH-start 3- <9 (n=53)	GH-start 9- (n=36)	p-value	Difference between groups Mean (95% CI)	Effect Size
**At adult height**					
**Age (yrs)**	16.8 (1.3)<br>16.5 (14.7; 21.4)<br>n=53	17.9 (1.5)<br>18 (14.8; 23.5)<br>n=36	0.0002	-1.16 (-1.74; -0.57)	0.843
**Adult height (cm)**	155.8 (6.0)<br>156.1 (145.1; 168.3)<br>n=53	157.8 (4.1)<br>158.1 (145.7; 165.5)<br>n=36	0.075	-2.08 (-4.31; 0.22)	0.391
**$Height_{SDS}$**	-1.95 (0.98)<br>-1.89 (-3.7; 0.12)<br>n=53	-1.60 (0.68)<br>-1.56 (-3.6; -0.34)<br>n=36	0.075	-0.341 (-0.709; 0.037)	0.391
**Diff MPH_{SDS}**	-1.70 (0.93)<br>-1.64 (-4.07; 0.49)<br>n=53	-1.45 (0.90)<br>-1.5 (-3.92; 0.03)<br>n=35	0.21	-0.252 (-0.648; 0.144)	0.275
**Gain in $height_{SDS}$ Puberty onset - Adult height**	-1.21 (0.43)<br>-1.21 (-2.08; -0.54)<br>n=53	-0.430 (0.556)<br>-0.478 (-1.781; 1.321)<br>n=36	<.0001	-0.778 (-0.984; -0.569)	1.61
**Gain in height (cm) Puberty onset - Adult height**	6.46 (5.19)<br>4.8 (-0.2; 20.5)<br>n=53	8.24 (4.99)<br>7.4 (1.1; 20.1)<br>n=36	0.12	-1.78 (-3.95; 0.48)	0.348
**Average GH $dose_{Total}$ (µg/kg/day)**	46.6 (12.5)<br>50 (31; 65.9)<br>n=53	42.8 (12.8)<br>34.6 (31.2; 63.4)<br>n=36	0.15	3.86 (-1.48; 9.27)	0.306
**Time on GH (yrs)**	9.88 (1.87)<br>9.86 (5.36; 13.87)<br>n=53	5.29 (1.55)<br>5.42 (2.62; 8.29)<br>n=36	<.0001	4.58 (3.83; 5.33)	2.62
**Duration puberty (yrs)**	3.10 (1.44)<br>3.03 (0.98; 9.26)<br>n=53	3.27 (1.46)<br>3.24 (0.98; 8.15)<br>n=36	0.61	-0.163 (-0.776; 0.478)	0.112
**Gain in $height_{SDS}$ GH start - Adult height**	0.883 (0.776)<br>0.817 (-0.694; 3.269)<br>n=53	1.22 (0.78)<br>1.4 (-1.32; 2.39)<br>n=36	0.048	-0.335 (-0.667; -0.004)	0.432

For continuous variables Mean (SD) / Median (Min; Max) / n= is presented.
For comparison between groups the Fisher´s Non Parametric Permutation Test was used for continuous variables.
The confidence interval for the mean difference between groups is based on Fishers non-parametric permutation test.
Effect size is absolute difference in mean / pooled SD.
GH, growth hormone; SDS, standard deviation score; MPH, midparental height; DiffMPH, difference in SD score between the height of the girl and the heights of her parents.

Table of Contents:

References: 2

Suppl Table 1a. Pre-treatment characteristics for the young subgroup versus dose in the PP population versus Swedish references {1,2,3,4]. 3

Suppl Table 1b. Auxology from GH-start for the young subgroup versus dose for the PP population versus Swedish references {1,2,3,4] 4

Suppl Table 1c Auxology at adult height for the young subgroup versus dose in the PP population versus the Swedish references [1,2,3,4].. 5

Suppl Table 2a. Pre-treatment characteristics for the old subgroup versus dose in the PP population versus Swedish references {1,2,3,4]. 6

Suppl Table 2b. Auxology from GH-start for the old subgroup versus dose for the PP population versus Swedish references {1,2,3,4] 7

Suppl Table 2c Auxology at adult height for the old subgroup versus dose in the PP population versus the Swedish references [1,2,3,4].. 8

Suppl Table 3a.Pre-treatment characteristics for all girls versus dose in the ITT population, versus Swedish references {1,2,3,4]. 9

Suppl Table 3b. Auxology from GH-start for all girls versus dose for the ITT population versus Swedish references {1,2,3,4]. 10

Suppl Table 3c Auxology at adult height for all girls versus dose 11

in the ITT population versus the Swedish references {1,2,3,4] 11

Suppl Table 4a. Pre-treatment characteristics for all girls versus age at GH-start in ITT population versus the Swedish references {1,2,3,4]. 12

Suppl Table 4b. Auxology from GH-start for all girls versus age at GH-start in the ITT population versus the Swedish references {1,2,3,4]. 13

Suppl Table 4c. Auxology at adult height for all girls versus age at GH-start in the ITT population versus the Swedish references {1,2,3,4].. 14

Suppl Table 5. Growth from birth to adult height for all girls versus age at GH-start and dose in the PP population; expressed in SDS versus the Turner reference 5,6] 15

Suppl Table 6a. Pre-treatment characteristics for all girls versus dose in the PP population, versus the Swedish references {I,2,3,4] 16

Suppl Table 6b. Auxology from GH-start for all girls versus dose in the PP population versus the Swedish references [1,2,3,4]. 17

Suppl Table 6c Auxology at adult height for all girls versus dose in the PP population versus the Swedish references [1,2,3,4].. 18

Suppl Table 7a. Pre-treatment characteristics for all girls versus age at GH-start in the PP population versus the Swedish reference {1,2,3,4]. 19

Suppl Table 7b. Auxology from GH-start for all girls versus age at GH-start in the PP population versus the Swedish references [1,2,3,4]. 20

Suppl Table 7c. Auxology at adult height for all girls versus age at GH-start in the PP population versus the Swedish references [1,2,3,4]. 21
